# First imported case of New World leishmaniasis in Romania: diagnostic and therapeutic challenges in a non-endemic country

**DOI:** 10.1186/s40249-026-01448-3

**Published:** 2026-05-02

**Authors:** Andrei Daniel Mihalca, Ioana Bianca Mitrea, Mihaela Sorina Lupșe, Angela Monica Ionică, Gad Baneth, Yaarit Nachum-Biala, Jaideep Kumar, Marian Taulescu, Filipe Dantas-Torres, Felipe Marinho Rocha de Macedo, Simona Corina Șenilă

**Affiliations:** 1 Department of Parasitology and Parasitic Diseases, University of Agricultural Sciences and Veterinary Medicine of Cluj-Napoca, Cluj-Napoca, Romania; 2 Department of Pharmacology, University of Agricultural Sciences and Veterinary Medicine of Cluj-Napoca, Cluj-Napoca, Romania; 3 Department of Infectious Diseases and Epidemiology, Iuliu Hațieganu University of Medicine and Pharmacy, Cluj-Napoca, Romania; 4 Clinical Hospital of Infectious Diseases of Cluj-Napoca, Cluj-Napoca, Romania; 5 Koret School of Veterinary Medicine, The Hebrew University of Jerusalem, Rehovot, Israel; 6 Department of Pathology, University of Agricultural Sciences and Veterinary Medicine of Cluj-Napoca, Cluj-Napoca, Romania; 7 Laboratory of Immunoparasitology, Department of Immunology, Aggeu Magalhães Institute, Oswaldo Cruz Foundation, Recife, Brazil; 8 Centro de Estudos Dermatológicos do Recife (CEDER), Santa Casa de Misericórdia do Recife, Recife, Brazil; 9 Department of Dermatology, Iuliu Hațieganu University of Medicine and Pharmacy, Cluj-Napoca, Romania; 10 Department of Dermatology, Emergency County Hospital, Cluj-Napoca, Romania

**Keywords:** New World leishmaniasis, *Leishmania panamensis*, Cutaneous leishmaniasis, Imported leishmaniasis, Romania

## Abstract

**Background:**

Leishmaniasis is a vector-borne protozoan disease with cutaneous, mucocutaneous, and visceral forms. In Europe, most cases involve Old World species, while imported New World infections are rare. We report the first imported human case of New World cutaneous leishmaniasis in Romania.

**Case presentation:**

A 25-year-old woman developed progressive ulcerative lesions on her left arm after travel to Panama and Costa Rica. Initial biopsy revealed granulomatous inflammation without identifying the agent. A second biopsy detected amastigotes within macrophages, and molecular analyses (ITS1 sequencing, *hsp70* phylogenetic analysis) identified *Leishmania panamensis*. The patient received oral miltefosine for 28 days, with gradual improvement and complete healing with atrophic scars. No relapse occurred 473 days post-treatment.

**Conclusions:**

This case highlights diagnostic challenges in non-endemic regions, emphasizing the need to raise clinical awareness and improve access to molecular tools for prompt recognition and management of imported leishmaniasis in Eastern Europe.

**Supplementary Information:**

The online version contains supplementary material available at 10.1186/s40249-026-01448-3.

## Background

Leishmaniasis is a sand fly-borne disease of humans and animals caused by more than 20 species of the genus *Leishmania*. In humans, three clinical forms are recognized, namely cutaneous, mucocutaneous, and visceral, all of which are considered neglected tropical diseases [1]. In humans, leishmaniasis is endemic in 98 countries across Asia, Africa, the Middle East, and Central and South America, where an estimated 12 million people are infected [2]. The annual incidence of cutaneous leishmaniasis is 700,000 to 1.2 million cases [1]. In Latin America, Central American countries account for 12% of the annual patient volume, with approximately 39,000 new cases reported each year [3]. A diverse range of species in the genus *Leishmania,* as well as different sand fly vector genera, are involved in the transmission in the Old (Eastern Hemisphere, particularly Afro-Eurasia) and New (Western Hemisphere, particularly the Americas) World [1, 2].

In addition to cases in endemic countries, imported cases are reported with an increased rate in various parts of the world [2]. This emergence of imported cases is related to several factors, all in connection with increased human movement (including migration) and globalization [2]. A recent review analysed a total of 9642 cases of non-endemic cutaneous leishmaniasis (CL) reported between 2000 and 2021, with only 32.7% of them being reported in travelers, while the remaining cases were reported in migrants or refugees [2]. The same study also analysed the probable country of infection, and cases from the New World represented less than 20% [2].

In southern Romania, autochthonous sporadic human cases of visceral leishmaniasis were reported between 1919 and 1955. Since then, only a few imported cases of visceral leishmaniasis have been reported in Romania [4, 5] all of which were caused by Old World species and originated from endemic European countries.

The present report presents the first case of New World leishmaniasis in Romania, together with its challenging diagnostics and treatment, due to limited awareness and laboratory capacity.

## Case presentation

**Fig. 1:**
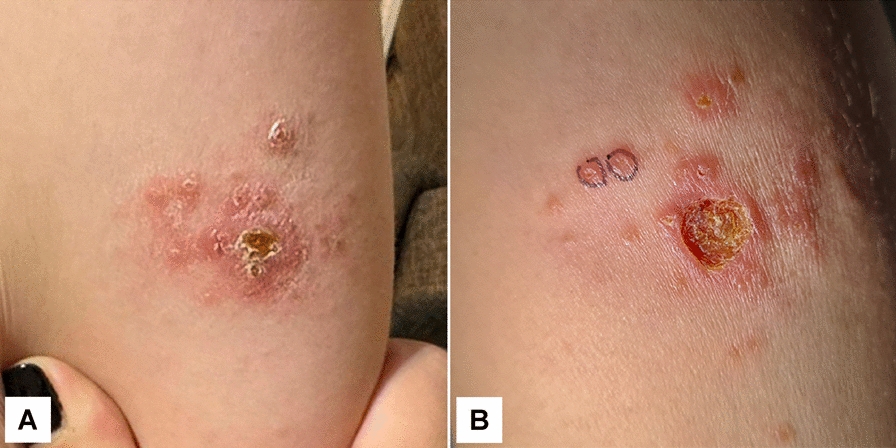
Initial stage of the lesions. **A** 4th of April 2024 (94 days since lesion onset). Erythematous infiltrated plaque with a central ulcer covered by yellowish-brown crust, surrounded by multiple satellite papules and nodules. **B** 10th of April 2024 (100 days since lesion onset). Progression of the lesion showing a well-defined ulcerative area with thick crust and persistent satellite papules in the surrounding skin

A 25-year-old female patient voluntarily presented to a dermatological consult in a private clinic from Cluj-Napoca on 10th of April 2024, due to progressive multifocal ulcerative lesions on the posterior brachial region of the left arm. According to the patient’s history, the lesions initially appeared as small, unpainful papules on January 1, 2024, which progressed over time (Fig. 1A) to an erythematous, infiltrated plaque with a central crusted ulcer and multiple satellite papules. The patient has reported a recent trip to northern Panama (Bocas del Toro) and southern Costa Rica (Puntarenas) between December 15 and 30, 2023. The first physical examination (10th of April 2024) revealed ulcerative lesions on the posterior aspect of the left arm (Fig. 1B). The primary lesion consisted of a well-defined ulcer covered by a thick yellowish-brown crust, surrounded by multiple smaller erythematous papules and nodules (one of which was selected for biopsy), indicating a relatively rapid progression within less than one week.

Following the physical examination, a list of differential diagnoses was provided, which included CL, atypical mycobacteriosis, and sporotrichosis, and a first punch biopsy was collected from two secondary satellite lesions (Fig. 1B—circled lesions).

Skin biopsy specimens were fixed in 10% neutral-buffered formalin, routinely processed, embedded in paraffin, and sectioned at 4 µm. Sections were stained with hematoxylin and eosin (H&E) for general histopathological evaluation. Additional special stains were performed to investigate possible infectious etiologies and differential diagnoses: Giemsa for detection of protozoal organisms, periodic acid-Schiff (PAS) for fungal elements, and Ziehl–Neelsen (ZN) for acid-fast bacilli.

The histopathological result of this first biopsy reported a cutaneous granulomatous lesion with unknown etiology. Giemsa staining showed the presence of a few mastocytes. PAS staining was positive in a few cells, revealing intracytoplasmic granules, but no spores, hyphae, or asteroid bodies were observed. Ziehl–Neelsen staining was positive in the same amphophilic cells, but no bacilli were detected.

**Fig. 2:**
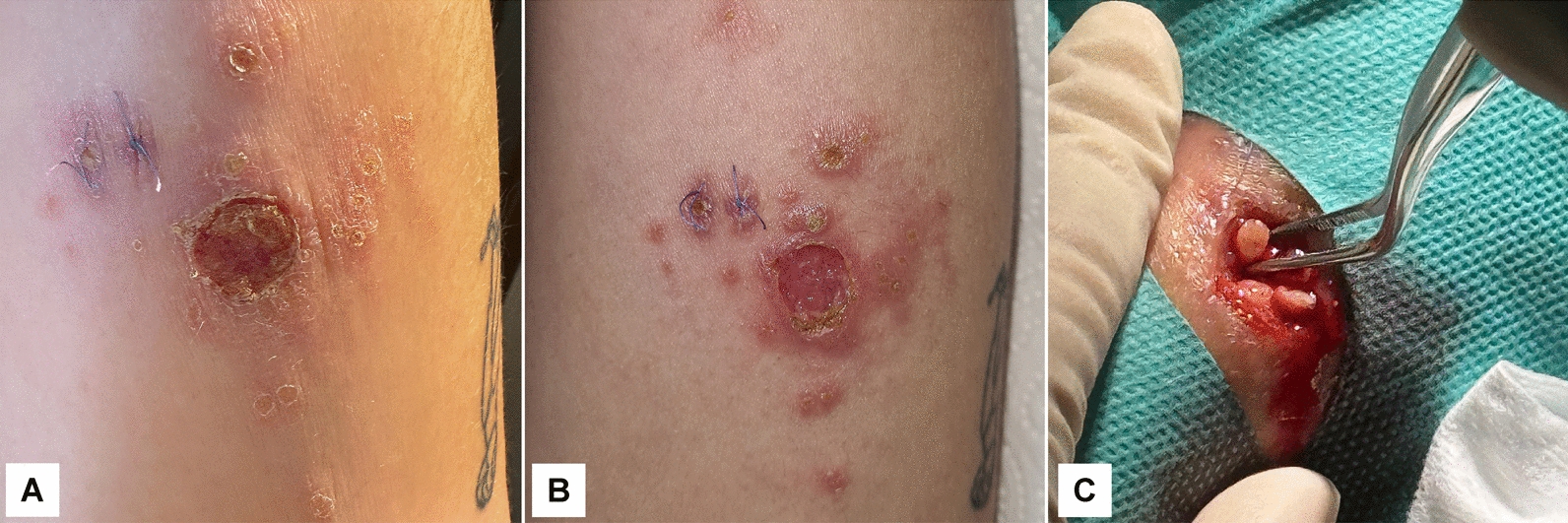
The cutaneous lesions and second biopsy on 24th April 2024 (114 days since lesion onset). **A** Ulcerative lesion with central crust and surrounding erythematous papules, with sutures from the first biopsy still visible. **B** The same lesion after cleaning of the ulcer base to remove debris, showing a better-defined fundus in preparation for biopsy. **C** Excision biopsy from the ulcer

As no definitive diagnosis was reached after the first biopsy, and the lesions continued to progress, a second biopsy was performed on 24th April 2024 (114 days since lesion onset). Concurrently, the ulcer on the posterior left arm had enlarged, featuring more clearly demarcated borders and increased surrounding erythema, while additional satellite papules became evident (Fig. 2A). After cleaning the ulcer base to remove debris and exposing the fundus (Fig. 2B), tissue was collected from the active margin of the lesion (Fig. 2C). Part of the tissue sample was processed for repeat histopathological examination. Another fragment, preserved in physiological saline solution, was submitted for DNA extraction, and further molecular diagnosis was performed at the Laboratory of Parasitology, Koret School of Veterinary Medicine, The Hebrew University of Jerusalem, Israel.

Leishmanial DNA was amplified using the internal transcribed spacer ITS1-PCR-high resolution melt (HRM) analysis. To increase the robustness of species identification, sequencing of the heat-shock protein 70 (*hsp70*) gene was also carried out, followed by phylogenetic analysis (details in Supplementary File 1) [6, 7]. DNA sequences were evaluated with the MEGA11 software [8], and compared for similarity with sequences available in GenBank®, using the BLAST program (http://www.ncbi.nlm.nih.gov/BLAST/). Sequences generated in this study were deposited in Genbank® under the following accession numbers PX864256 and PX872204.

Phylogenetic relationships were inferred based on 36 *Leishmania* spp. nucleotide sequences using IQ-TREE 3.0.1 (http://www.iqtree.org) [9, 10]. The dataset comprised 1,088 nucleotide sites, of which 91.73% were constant. Based on the Bayesian Information Criterion (BIC), the Tamura-Nei + F + I (base frequencies and invariable sites) substitution model was selected via ModelFinder [11, 12]. Branch support was assessed using 10,000 ultrafast bootstrap replicates.

**Fig. 3:**
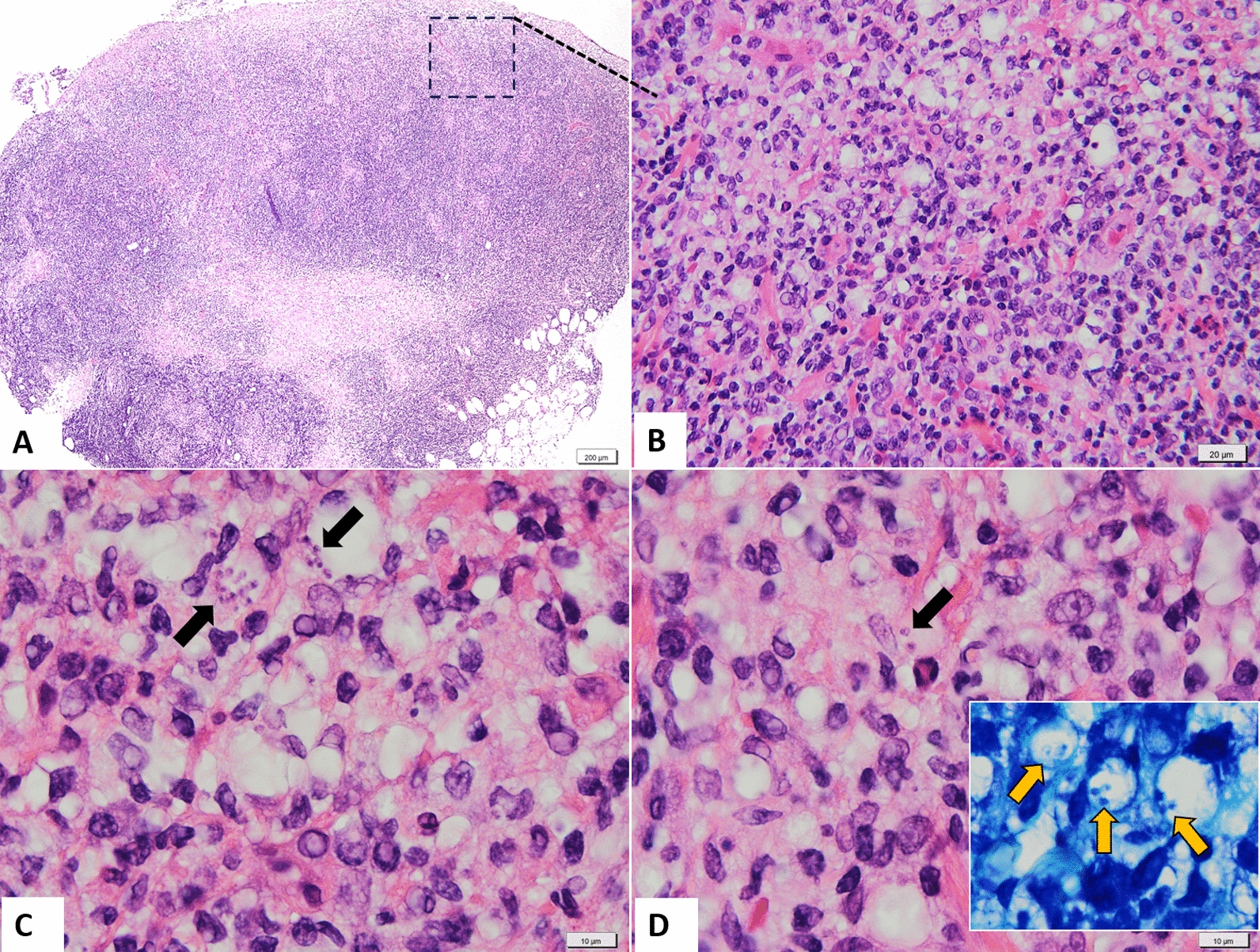
Histological findings (H&E stain). **A** General aspect of the ulcerated cutaneous nodules. **B** Severe histiocytic and lymphoplasmacytic inflammation in the dermis. **C** Intrahistiocytic amastigotes of *Leishmania* spp. (black arrows). **D** Amastigotes (black arrows) with details of infected macrophages (yellow arrows) (inset with Giemsa stain)

The second histopathological examination demonstrated severe and diffuse necrosis and ulceration of the epidermis and superficial papillary dermis (Fig. 3A). The reticular deep dermis and subcutis were severely effaced and infiltrated by large numbers of macrophages, small lymphocytes, and plasma cells, admixed with a few Mott cells and eosinophils (Fig. 3A, B). In the ulcerated areas, hemorrhages, oedema, congestion, and neutrophil infiltrates were also noticed. Multifocal, the macrophages contained intracytoplasmic 3–4 µm round-oval structures morphologically similar to *Leishmania* spp. amastigotes, with 1–2 µm diameter basophilic nucleus and a perpendicular kinetoplastid (Fig. 3C, D).

The ITS1 sequence obtained from the skin lesion (accession number PX864256) was 100% identical to both *L. panamensis* (e.g., isolate J237, GenBank MT606243.1) and *L. guyanensis* (e.g., isolate J176, GenBank MT606266.1), confirming its placement within the *Viannia* subgenus and *L*. *guyanensis* complex. The *hsp70* sequence from the skin lesion (accession number PX872204) had 99.9% identity to *L. panamensis* (ON806904.1). Gel showing the PCR results of the *hsp70* PCR is shown in Supplementary File 2. The *hsp70* phylogenetic analysis provided higher discriminatory power and clustered the isolate unambiguously with reference sequences of *L. panamensis*, supported by robust bootstrap values (Supplementary File 2).

**Fig. 4:**
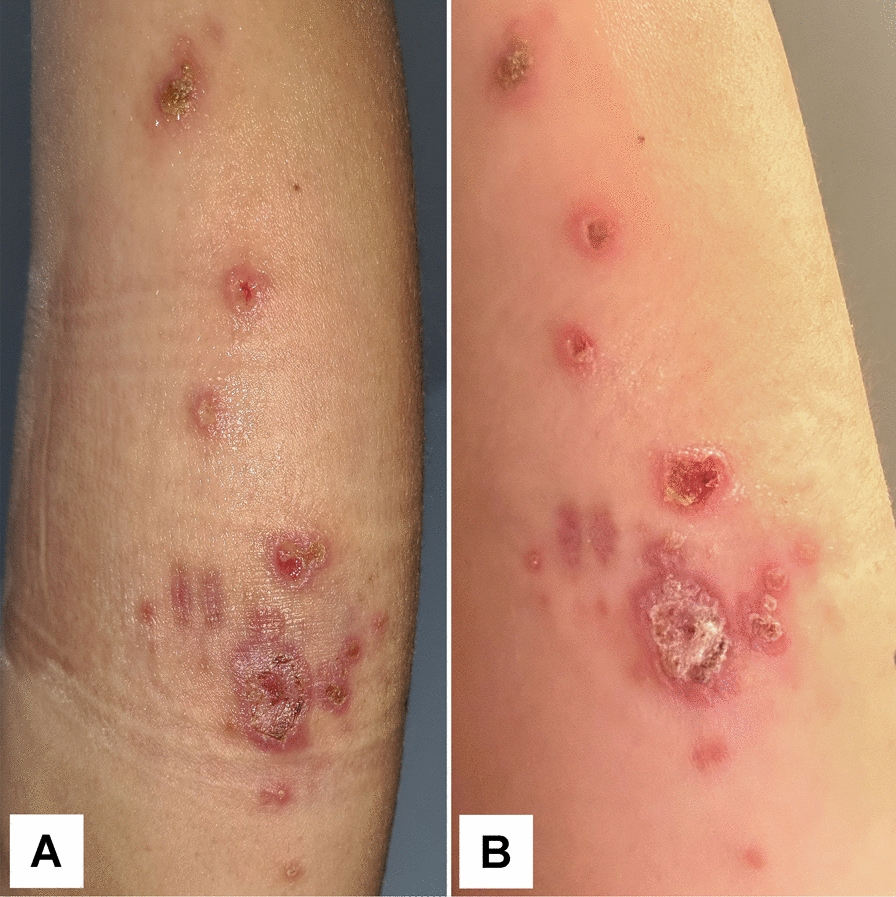
The cutaneous lesions before the treatment was initiated. **A** 13th May 2024 (133 days since lesion onset). The central ulcer with overlying crust, surrounded by multiple smaller erythematous papules and nodules, some with superficial ulceration. **B** 18th May 2024 (138 days since lesion onset). Further progression of the lesions, with increased inflammatory reaction and persistence of both the main ulcer and satellite papules

By mid-May 2024, the lesions had not improved and showed further progression. On 13th May 2024 (133 days since lesion onset), the main ulcer on the posterior arm remained covered by a thick crust, with multiple smaller erythematous papules and nodules in the surrounding skin, some of which had begun to ulcerate (Fig. 4A). Five days later, on 18th May 2024 (138 days since lesion onset), the lesions persisted with a more intense inflammatory halo and continued presence of satellite papules (Fig. 4B). These findings indicated an active, progressive cutaneous process, and treatment was initiated shortly thereafter.

On 20th May 2024 (140 days since lesion onset), treatment with miltefosine Impavido®, one 50 mg capsule, three times daily, i.e. every 8 h was initiated after the drug was imported from Brazil (the drug is not available in Romania). During treatment, the patient experienced gastrointestinal (nausea, vomiting, diarrhea, and decreased appetite) and systemic (headache, dizziness, general weakness, and malaise) side effects. The treatment was concluded on 16th June 2024.

**Fig. 5:**
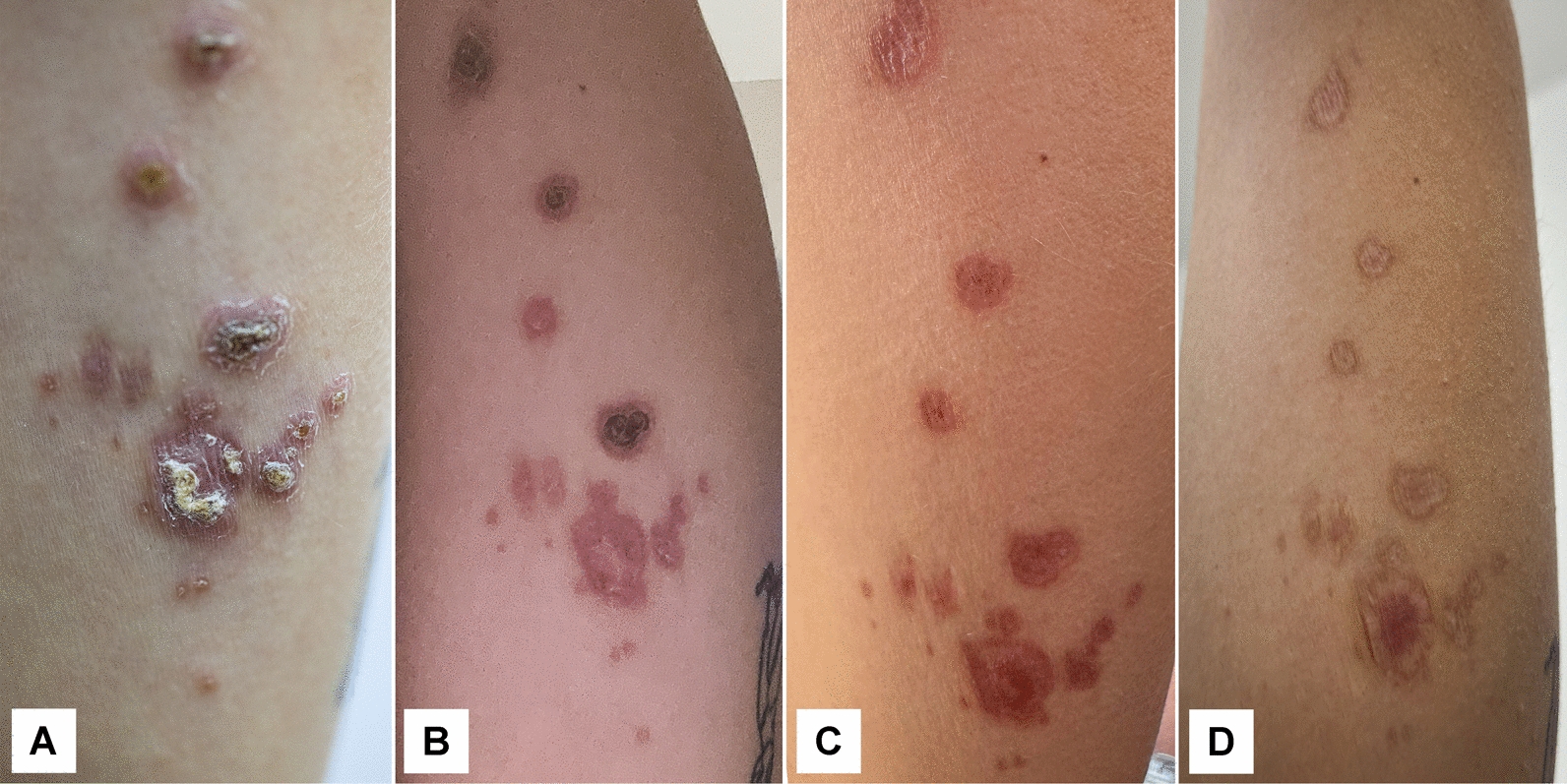
The progression of lesions following treatment initiation. **A** 30th May 2024 (day 10 after the start of treatment), partial re-epithelialization of the ulcer and reduction of crusting were observed. **B** 13th June 2024 (day 24 after the start of treatment), lesions showed further healing with decreased inflammatory halo and regression of satellite papules. **C** 9th July 2024 (23 days since treatment end), the ulcer had almost completely resolved, leaving post-inflammatory changes. **D** 13th November 2024 (150 days since treatment end), five months after treatment initiation, the lesions had fully healed, leaving atrophic scars

**Fig. 6:**
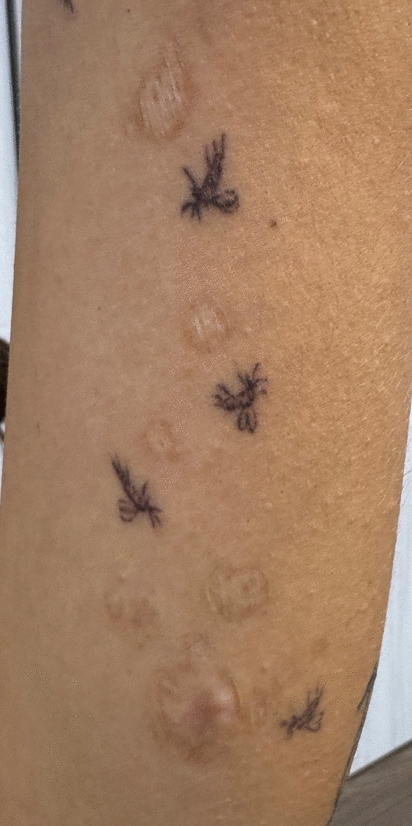
Long-term outcome at follow-up on 5th September 2025 (446 days since treatment end). Multiple atrophic scars are visible, corresponding to the sites of previous ulcerative lesions. As a personal reminder of the experience, the patient chose to surround the scars with small sand fly tattoos

The therapeutic response was favorable, with clear evidence of progressive healing after the initiation of miltefosine. After 10 days of antiparasitic treatment, partial re-epithelialization and reduction of crusting were already visible. Near the end of therapy, on day 24, the ulcer showed further contraction, diminished inflammatory reaction, and disappearance of most satellite papules. By day 50, several weeks after treatment completion, the lesion had nearly resolved, leaving only residual post-inflammatory changes. At follow-up on day 177, complete healing had occurred, with the formation of permanent atrophic scars (Fig. 5). At 446 days after the treatment ended, no relapse was observed, but permanent scars remained (Fig. 6).

## Discussion and conclusions

Imported cases of CL have been increasingly reported in non-endemic European countries over the last two decades, reflecting global travel, migration, and changing epidemiological patterns. In Central Europe, Austria documented 340 cases between 2000 and 2021 [13]. The vast majority were caused by Old World species, while only three were attributed to New World species: two cases due to *L. braziliensis* (one acquired in French Guiana and one in Bolivia) and one case caused by *L. panamensis* (imported from Costa Rica). In Poland, 14 cases of imported CL were reported between 2005 and 2017, including seven acquired in Latin America (Belize, Cuba, French Guiana, Bolivia, and Peru). However, in all of these, the *Leishmania* species involved remained unidentified [14].

This report presents the first case of New World CL in Romania, along with its challenging diagnosis and treatment, due to limited awareness and laboratory capacity. Similar diagnostic difficulties have been highlighted in other non-endemic settings. In Poland, despite the often typical clinical appearance of lesions, the time to correct diagnosis was frequently prolonged, underlining a lack of awareness among physicians and the need to improve access to diagnostic methods appropriate for CL [14]. In Slovakia, a traveler infected with *L. panamensis* initially had negative results from wound scraping and biopsy, and the diagnosis was confirmed only after repeated sampling and subsequent molecular analysis [15]. At a broader scale, a global review of imported leishmaniases [2] underscored that non-endemic regions often face challenges in selecting and applying adequate diagnostic tools, with species identification and tailored treatment strategies frequently hampered by laboratory limitations.

In Romania, there are nine species of sand flies reported, some of them only with decades old records (*Phlebotomus alexandri*, *P. longiductus*), and other reported also recently (*P. neglectus*, *P. perfiliewi*, *P. papatasi*, *P. balcanicus, P. sergenti*, *P. simonahalepae*, and *Sergentomyia minuta* [16–18]. Although some of these species are known as competent vectors for Old World *Leishmania* species (i.e. *P. neglectus*, *P. perfiliewi*, and *P. balcanicus* for *L. infantum*), their vectorial role for New World *Leishmania* is limited to experimental studies. Most sand fly species tested to date, including members of the *Larroussius* and *Adlerius* subgenera, belong to the category of permissive vectors [19]. This characteristic means that they support the development of multiple *Leishmania* species under experimental conditions and allow the parasites to mature in the sandflies’ midguts [20, 21], but the risk of introduction and establishment of New World *Leishmania* species to Europe remains unlikely.

An additional therapeutic challenge in our case was the unavailability of miltefosine in Romania, which necessitated its importation from abroad. This delay and difficulty in accessing effective anti-*Leishmania* therapy further illustrate the vulnerability of patients in non-endemic countries, where both diagnostic and therapeutic resources for leishmaniasis are scarce.

Our case represents the first documented instance of New World CL in Romania, highlighting the diagnostic challenges that arise in non-endemic regions. The rarity of such cases in Central and Eastern Europe, the frequent overlap of clinical manifestations with more common conditions, and the limited availability of species-level diagnostics all contribute to delayed or missed diagnosis. Strengthening clinician awareness and ensuring access to advanced diagnostic tools are therefore essential for improving the recognition and management of imported leishmaniasis in this region.

## Supplementary Information

Additional file 1.

Additional file 2.

## Data Availability

All data and materials analysed during this study are included in this published article.
