# Supplementary material for: First imported case of New World leishmaniasis in Romania: diagnostic and therapeutic challenges in a non-endemic country
Source: Infect Dis Poverty. 2026 May 2;15:47. doi: 10.1186/s40249-026-01448-3 (PMC13135252; doi:10.1186/s40249-026-01448-3)
Supplement: Supplementary file 1 — Additional file 1. [file 40249_2026_1448_MOESM1_ESM.docx]

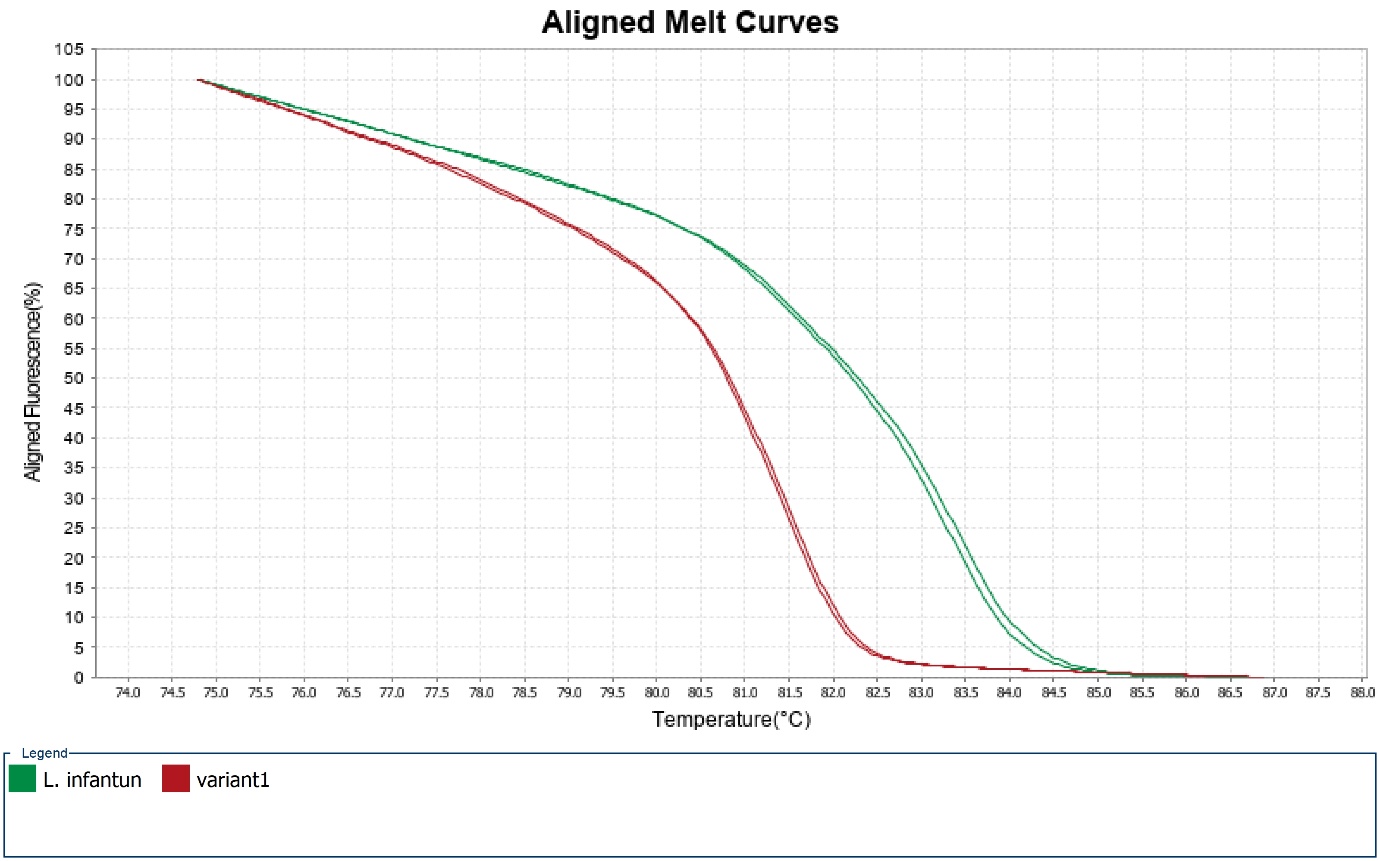


HRM plot of sample I-1-s (red line) and *Leishmania infantum* (green line)

Sequencing of the heat-shock protein 70 (*hsp70*) gene was carried out using primes HSP70-F25/ HSP70-R1310. Positive PCR products were sequenced using both forward and reverse primes using the BigDye Terminator v3.1 Cycle Sequencing Kit and an ABI PRISM 3100 Genetic Analyzer (Applied Biosystems, Foster City, CA, USA), at the Centre for Genomic Technologies, Hebrew University of Jerusalem, Israel.
