# Supplementary material for: First imported case of New World leishmaniasis in Romania: diagnostic and therapeutic challenges in a non-endemic country
Source: Infect Dis Poverty. 2026 May 2;15:47. doi: 10.1186/s40249-026-01448-3 (PMC13135252; doi:10.1186/s40249-026-01448-3)
Supplement: Supplementary file 2 — Additional file 2. [file 40249_2026_1448_MOESM2_ESM.pdf]

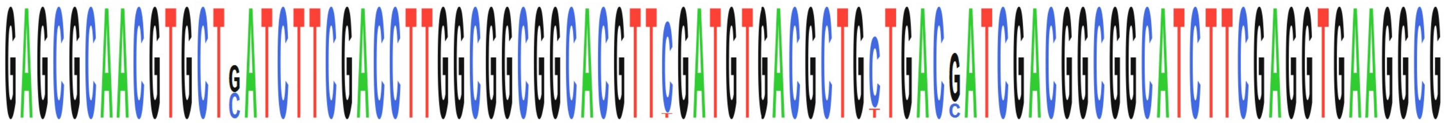

**Consensus**	GAGCGCAACGTGCTGATCTTCGACCTTGGCGGCGGCACGTTCGATGTGACGCTGCTGACGATCGACGGCGGCATCTTCGAGGTGAAGGCG	
L. Panamensis_hsp70	GAGCGCAACGTGCTCATCTTCGACCTTGGCGGCGGCACGTTCGATGTGACGCTGCTGACGATCGACGGCGGCATCTTCGAGGTGAAGGCG	90
EU599094_L. Pan...is_hsp70_Panama	GAGCGCAACGTGCTCATCTTCGACCTTGGCGGCGGCACGTTCGATGTGACGCTGCTGACGATCGACGGCGGCATCTTCGAGGTGAAGGCG	90
FN395055_L. Pan...is_hsp70_Panama	GAGCGCAACGTGCTCATCTTCGACCTTGGCGGCGGCACGTTCGATGTGACGCTGCTGACGATCGACGGCGGCATCTTCGAGGTGAAGGCG	90
KX573937_L. Pan...is_hsp70_Panama	GAGCGCAACGTGCTCATCTTCGACCTTGGCGGCGGCACGTTCGATGTGACGCTGCTGACGATCGACGGCGGCATCTTCGAGGTGAAGGCG	90
KX573939_L. Pan...is_hsp70_Panama	GAGCGCAACGTGCTCATCTTCGACCTTGGCGGCGGCACGTTCGATGTGACGCTGCTGACGATCGACGGCGGCATCTTCGAGGTGAAGGCG	90
KX573942_L. Pan...is_hsp70_Panama	GAGCGCAACGTGCTCATCTTCGACCTTGGCGGCGGCACGTTCGATGTGACGCTGCTGACGATCGACGGCGGCATCTTCGAGGTGAAGGCG	90
KX573971_L. Pan...is_hsp70_Panama	GAGCGCAACGTGCTCATCTTCGACCTTGGCGGCGGCACGTTCGATGTGACGCTGCTGACGATCGACGGCGGCATCTTCGAGGTGAAGGCG	90
KX573975_L. Pan...is_hsp70_Panama	GAGCGCAACGTGCTCATCTTCGACCTTGGCGGCGGCACGTTCGATGTGACGCTGCTGACGATCGACGGCGGCATCTTCGAGGTGAAGGCG	90
OQ408896_L. Pan...is_hsp70_Panama	GAGCGCAACGTGCTCATCTTCGACCTTGGCGGCGGCACGTTCGATGTGACGCTGCTGACGATCGACGGCGGCATCTTCGAGGTGAAGGCG	90
MW480568_L. Pa...s_hsp70_Norway	GAGCGCAACGTGCTCATCTTCGACCTTGGCGGCGGCACGTTCGATGTGACGCTGCTGACGATCGACGGCGGCATCTTCGAGGTGAAGGCG	90
HF586406_L. guy...0_French Guiana	GAGCGCAACGTGCTCATCTTCGACCTTGGCGGCGGCACGTTCGATGTGACGCTGCTGACGATCGACGGCGGCATCTTCGAGGTGAAGGCG	90
KX573934_L. guy...is_hsp70_Panama	GAGCGCAACGTGCTCATCTTCGACCTTGGCGGCGGCACGTTCGATGTGACGCTGCTGACGATCGACGGCGGCATCTTCGAGGTGAAGGCG	90
KX574011_L. guy...is_hsp70_Panama	GAGCGCAACGTGCTCATCTTCGACCTTGGCGGCGGCACGTTCGATGTGACGCTGCTGACGATCGACGGCGGCATCTTCGAGGTGAAGGCG	90
MW658405_ L. gu...70_isolate_PL11	GAGCGCAACGTGCTCATCTTCGACCTTGGCGGCGGCACGTTCGATGTGACGCTGCTGACGATCGACGGCGGCATCTTCGAGGTGAAGGCG	90
MW658386_ L. gu...70_isolate_PL21	GAGCGCAACGTGCTCATCTTCGACCTTGGCGGCGGCACGTTCGATGTGACGCTGCTGACGATCGACGGCGGCATCTTCGAGGTGAAGGCG	90
MW658403_ L. gu...p70_isolate_PL6	GAGCGCAACGTGCTCATCTTCGACCTTGGCGGCGGCACGTTCGATGTGACGCTGCTGACGATCGACGGCGGCATCTTCGAGGTGAAGGCG	90
MG029123_L. ama...is_hsp70_Brazil	GAGCGCAACGTGCTGATCTTCGACCTTGGCGGCGGCACGTTCGATGTGACGCTGCTGACCATCGACGGCGGCATCTTCGAGGTGAAGGCG	90
EU599090_L. amaz...sis_hsp70_Brazil	GAGCGCAACGTGCTGATCTTCGACCTTGGCGGCGGCACGTTCGATGTGACGCTGCTGACCATCGACGGCGGCATCTTCGAGGTGAAGGCG	90
MF344859_L. ama...is_hsp70_Brazil	GAGCGCAACGTGCTGATCTTCGACCTTGGCGGCGGCACGTTCGATGTGACGCTGCTGACCATCGACGGCGGCATCTTCGAGGTGAAGGCG	90
MF344857_L. ama...is_hsp70_Brazil	GAGCGCAACGTGCTGATCTTCGACCTTGGCGGCGGCACGTTCGATGTGACGCTGCTGACCATCGACGGCGGCATCTTCGAGGTGAAGGCG	90
OP561801_L. amaz...is_hsp70_Bolivia	GAGCGCAACGTGCTGATCTTCGACCTTGGCGGCGGCACGTTCGATGTGACGCTGCTGACCATCGACGGCGGCATCTTCGAGGTGAAGGCG	90
LN907831_L. ama...0_French Guiana	GAGCGCAACGTGCTGATCTTCGACCTTGGCGGCGGCACGTTCGATGTGACGCTGCTGACCATCGACGGCGGCATCTTCGAGGTGAAGGCG	90
FN395039_L. braz...is_hsp70_Bolivia	GAGCGCAACGTGCTCATCTTCGACCTTGGCGGCGGCACGTTCGATGTGACGCTGCTGACGATCGACGGCGGCATCTTCGAGGTGAAGGCG	90
FN395041_L. braz...is_hsp70_Bolivia	GAGCGCAACGTGCTCATCTTCGACCTTGGCGGCGGCACGTTCGATGTGACGCTGCTGACGATCGACGGCGGCATCTTCGAGGTGAAGGCG	90
FR872760_L. braz...is_hsp70_Bolivia	GAGCGCAACGTGCTCATCTTCGACCTTGGCGGCGGCACGTTCGATGTGACGCTGCTGACGATCGACGGCGGCATCTTCGAGGTGAAGGCG	90
KX573945_L. bra...is_hsp70_Panama	GAGCGCAACGTGCTCATCTTCGACCTTGGCGGCGGCACGTTCGATGTGACGCTGCTGACGATCGACGGCGGCATCTTCGAGGTGAAGGCG	90
FR872763_L. bra...nsis_hsp70_Peru	GAGCGCAACGTGCTCATCTTCGACCTTGGCGGCGGCACGTTCGATGTGACGCTGCTGACGATCGACGGCGGCATCTTCGAGGTGAAGGCG	90
HF586401_L. mex...na_hsp70_Mexico	GAGCGCAACGTGCTGATCTTCGACCTTGGCGGCGGCACGTTCGATGTGACGCTGCTGACCATCGACGGCGGCATCTTCGAGGTGAAGGCG	90
HF586413_L. mex...a_hsp70_Ecuador	GAGCGCAACGTGCTGATCTTCGACCTTGGCGGCGGCACGTTCGATGTGACGCTGCTGACCATCGACGGCGGCATCTTCGAGGTGAAGGCG	90
FN395038_ L. mexicana_hsp70_Peru	GAGCGCAACGTGCTGATCTTCGACCTTGGCGGCGGCACGTTCGATGTGACGCTGCTGACCATCGACGGCGGCATCTTCGAGGTGAAGGCG	90
EU599091_ L. me...a_hsp70_ Belize	GAGCGCAACGTGCTGATCTTCGACCTTGGCGGCGGCACGTTCGATGTGACGCTGCTGACCATCGACGGCGGCATCTTCGAGGTGAAGGCG	90
MN728741_L. don...i_ hsp70_ India	GAGCGCAACGTGCTGATCTTCGACCTTGGCGGCGGCACGTTCGATGTGACGCTGTTGACGATCGACGGCGGCATCTTCGAGGTGAAGGCG	90
MN728759_L. don...i_ hsp70_ Sudan	GAGCGCAACGTGCTGATCTTCGACCTTGGCGGCGGCACGTTCGATGTGACGCTGTTGACGATCGACGGCGGCATCTTCGAGGTGAAGGCG	90
MN728753_L. inf...m_ hsp70_ Malta	GAGCGCAACGTGCTGATCTTCGACCTTGGCGGCGGCACGTTCGATGTGACGCTGTTGACGATCGACGGCGGCATCTTCGAGGTGAAGGCG	90
MN728756_L. inf...m_ hsp70_ Sudan	GAGCGCAACGTGCTGATCTTCGACCTTGGCGGCGGCACGTTCGATGTGACGCTGTTGACGATCGACGGCGGCATCTTCGAGGTGAAGGCG	90
FN395032_L. infa... hsp70_ Portugal	GAGCGCAACGTGCTGATCTTCGACCTTGGCGGCGGCACGTTCGATGTGACGCTGTTGACGATCGACGGCGGCATCTTCGAGGTGAAGGCG	90
L. Tropica_hsp70	GAGCGCAACGTGCTGATCTTCGACCTTGGCGGCGGCACGTTCGATGTGACGCTGCTGACGATCGACGGCGGCATCTTCGAGGTGAAGGCG	90
LN907846_L. Tropica_hsp70_Israel	GAGCGCAACGTGCTGATCTTCGACCTTGGCGGCGGCACGTTCGATGTGACGCTGCTGACGATCGACGGCGGCATCTTCGAGGTGAAGGCG	90
MW658424_L. Tro...70_isolate_L192	GAGCGCAACGTGCTGATCTTCGACCTTGGCGGCGGCACGTTCGATGTGACGCTGCTGACGATCGACGGCGGCATCTTCGAGGTGAAGGCG	90
MW658401_L. Tro...p70_isolate_PL5	GAGCGCAACGTGCTGATCTTCGACCTTGGCGGCGGCACGTTCGATGTGACGCTGCTGACGATCGACGGCGGCATCTTCGAGGTGAAGGCG	90
MZ362273_L. Tro...p70_Afghanistan	GAGCGCAACGTGCTGATCTTCGACCTTGGCGGCGGCACGTTCGATGTGACGCTGCTGACGATCGACGGCGGCATCTTCGAGGTGAAGGCG	90
FN395023_L. major_ hsp70_Spain	GAGCGCAACGTGCTGATCTTCGACCTTGGCGGCGGCACGTTTGATGTGACGCTGCTGACGATCGACGGCGGCATCTTCGAGGTGAAGGCG	90
FN395022_ L. major_ hsp70_Sudan	GAGCGCAACGTGCTGATCTTCGACCTTGGCGGCGGCACGTTTGATGTGACGCTGCTGACGATCGACGGCGGCATCTTCGAGGTGAAGGCG	90

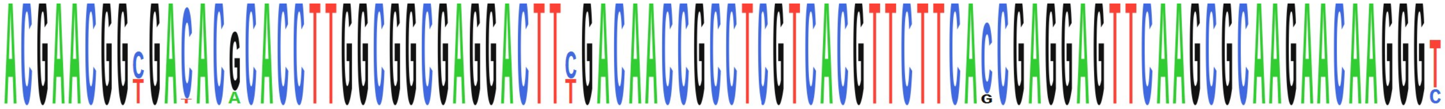

Sequence	Alignment	
**Consensus**	ACGAACGGCGACACGCACCTTGGCGGCGAGGACTTCGACAACCGCCTCGTCACGTTCTTCACCGAGGAGTTCAAGCGCAAGAACAAGGGT	
L. Panamensis_hsp70	ACGAACGGTGACACGCACCTTGGCGGCGAGGACTTTGACAACCGCCTCGTCACGTTCTTCACCGAGGAGTTCAAGCGCAAGAACAAGGGT	180
EU599094_L. Pan...is_hsp70_Panama	ACGAACGGTGACACGCACCTTGGCGGCGAGGACTTTGACAACCGCCTCGTCACGTTCTTCACCGAGGAGTTCAAGCGCAAGAACAAGGGT	180
FN395055_L. Pan...is_hsp70_Panama	ACGAACGGTGACACGCACCTTGGCGGCGAGGACTTTGACAACCGCCTCGTCACGTTCTTCACCGAGGAGTTCAAGCGCAAGAACAAGGGT	180
KX573937_L. Pan...is_hsp70_Panama	ACGAACGGTGACACGCACCTTGGCGGCGAGGACTTTGACAACCGCCTCGTCACGTTCTTCACCGAGGAGTTCAAGCGCAAGAACAAGGGT	180
KX573939_L. Pan...is_hsp70_Panama	ACGAACGGTGACACGCACCTTGGCGGCGAGGACTTTGACAACCGCCTCGTCACGTTCTTCACCGAGGAGTTCAAGCGCAAGAACAAGGGT	180
KX573942_L. Pan...is_hsp70_Panama	ACGAACGGTGACACGCACCTTGGCGGCGAGGACTTTGACAACCGCCTCGTCACGTTCTTCACCGAGGAGTTCAAGCGCAAGAACAAGGGT	180
KX573971_L. Pan...is_hsp70_Panama	ACGAACGGTGACACGCACCTTGGCGGCGAGGACTTTGACAACCGCCTCGTCACGTTCTTCACCGAGGAGTTCAAGCGCAAGAACAAGGGT	180
KX573975_L. Pan...is_hsp70_Panama	ACGAACGGTGACACGCACCTTGGCGGCGAGGACTTTGACAACCGCCTCGTCACGTTCTTCACCGAGGAGTTCAAGCGCAAGAACAAGGGT	180
OQ408896_L. Pan...is_hsp70_Panama	ACGAACGGTGACACGCACCTTGGCGGCGAGGACTTTGACAACCGCCTCGTCACGTTCTTCACCGAGGAGTTCAAGCGCAAGAACAAGGGT	180
MW480568_L. Pa...s_hsp70_Norway	ACGAACGGTGACACGCACCTTGGCGGCGAGGACTTTGACAACCGCCTCGTCACGTTCTTCACCGAGGAGTTCAAGCGCAAGAACAAGGGT	180
HF586406_L. guy...0_French Guiana	ACGAACGGTGACACGCACCTTGGCGGCGAGGACTTTGACAACCGCCTCGTCACGTTCTTCACCGAGGAGTTCAAGCGCAAGAACAAGGGT	180
KX573934_L. guy...is_hsp70_Panama	ACGAACGGTGACACGCACCTTGGCGGCGAGGACTTTGACAACCGCCTCGTCACGTTCTTCACCGAGGAGTTCAAGCGCAAGAACAAGGGT	180
KX574011_L. guy...is_hsp70_Panama	ACGAACGGTGACACGCACCTTGGCGGCGAGGACTTTGACAACCGCCTCGTCACGTTCTTCACCGAGGAGTTCAAGCGCAAGAACAAGGGT	180
MW658405_ L. gu...70_isolate_PL11	ACGAACGGTGACACGCACCTTGGCGGCGAGGACTTTGACAACCGCCTCGTCACGTTCTTCACCGAGGAGTTCAAGCGCAAGAACAAGGGT	180
MW658386_ L. gu...70_isolate_PL21	ACGAACGGTGACACGCACCTTGGCGGCGAGGACTTTGACAACCGCCTCGTCACGTTCTTCACCGAGGAGTTCAAGCGCAAGAACAAGGGT	180
MW658403_ L. gu...p70_isolate_PL6	ACGAACGGTGACACGCACCTTGGCGGCGAGGACTTTGACAACCGCCTCGTCACGTTCTTCACCGAGGAGTTCAAGCGCAAGAACAAGGGT	180
MG029123_L. ama...is_hsp70_Brazil	ACGAACGGCGACACGCACCTTGGCGGCGAGGACTTCGACAACCGCCTCGTCACGTTCTTCACCGAGGAGTTCAAGCGCAAGAACAAGGGC	180
EU599090_L. amaz...sis_hsp70_Brazil	ACGAACGGCGACACGCACCTTGGCGGCGAGGACTTCGACAACCGCCTCGTCACGTTCTTCACCGAGGAGTTCAAGCGCAAGAACAAGGGC	180
MF344859_L. ama...is_hsp70_Brazil	ACGAACGGCGACACGCACCTTGGCGGCGAGGACTTCGACAACCGCCTCGTCACGTTCTTCACCGAGGAGTTCAAGCGCAAGAACAAGGGC	180
MF344857_L. ama...is_hsp70_Brazil	ACGAACGGCGACACGCACCTTGGCGGCGAGGACTTCGACAACCGCCTCGTCACGTTCTTCACCGAGGAGTTCAAGCGCAAGAACAAGGGC	180
OP561801_L. amaz...is_hsp70_Bolivia	ACGAACGGCGACACGCACCTTGGCGGCGAGGACTTCGACAACCGCCTCGTCACGTTCTTCACCGAGGAGTTCAAGCGCAAGAACAAGGGC	180
LN907831_L. ama...0_French Guiana	ACGAACGGCGACACGCACCTTGGCGGCGAGGACTTCGACAACCGCCTCGTCACGTTCTTCACCGAGGAGTTCAAGCGCAAGAACAAGGGC	180
FN395039_L. braz...is_hsp70_Bolivia	ACGAACGGTGACACGCACCTTGGCGGCGAGGACTTTGACAACCGCCTCGTCACGTTCTTCAGCGAGGAGTTCAAGCGCAAGAACAAGGGT	180
FN395041_L. braz...is_hsp70_Bolivia	ACGAACGGTGACACGCACCTTGGCGGCGAGGACTTTGACAACCGCCTCGTCACGTTCTTCAGCGAGGAGTTCAAGCGCAAGAACAAGGGT	180
FR872760_L. braz...is_hsp70_Bolivia	ACGAACGGTGACACRCACCTTGGCGGCGAGGACTTTGACAACCGCCTCGTCACGTTCTTCAGCGAGGAGTTCAAGCGCAAGAACAAGGGT	180
KX573945_L. bra...is_hsp70_Panama	ACGAACGGTGACACGCACCTTGGCGGCGAGGACTTTGACAACCGCCTCGTCACGTTCTTCAGCGAGGAGTTCAAGCGCAAGAACAAGGGT	180
FR872763_L. bra...nsis_hsp70_Peru	ACGAACGGTGACACRCACCTTGGCGGCGAGGACTTTGACAACCGCCTCGTCACGTTCTTCAGCGAGGAGTTCAAGCGCAAGAACAAGGGT	180
HF586401_L. mex...na_hsp70_Mexico	ACGAACGGCGACACGCACCTTGGCGGCGAGGACTTCGACAACCGCCTCGTCACGTTCTTCACCGAGGAGTTCAAGCGCAAGAACAAGGGC	180
HF586413_L. mex...a_hsp70_Ecuador	ACGAACGGCGACACGCACCTTGGCGGCGAGGACTTCGACAACCGCCTCGTCACGTTCTTCACCGAGGAGTTCAAGCGCAAGAACAAGGGC	180
FN395038_ L. mexicana_hsp70_Peru	ACGAACGGCGACACGCACCTTGGCGGCGAGGACTTCGACAACCGCCTCGTCACGTTCTTCACCGAGGAGTTCAAGCGCAAGAACAAGGGC	180
EU599091_ L. me...a_hsp70_ Belize	ACGAACGGCGACACGCACCTTGGCGGCGAGGACTTCGACAACCGCCTCGTCACGTTCTTCACCGAGGAGTTCAAGCGCAAGAACAAGGGC	180
MN728741_L. don...i_ hsp70_ India	ACGAACGGCGACACGCACCTTGGCGGCGAGGACTTCGACAACCGCCTCGTCACGTTCTTCACCGAGGAGTTCAAGCGCAAGAACAAGGGT	180
MN728759_L. don...i_ hsp70_ Sudan	ACGAACGGCGACACGCACCTTGGCGGCGAGGACTTCGACAACCGCCTCGTCACGTTCTTCACCGAGGAGTTCAAGCGCAAGAACAAGGGT	180
MN728753_L. inf...m_ hsp70_ Malta	ACGAACGGCGACACGCACCTTGGCGGCGAGGACTTCGACAACCGCCTCGTCACGTTCTTCACCGAGGAGTTCAAGCGCAAGAACAAGGGT	180
MN728756_L. inf...m_ hsp70_ Sudan	ACGAACGGCGACACGCACCTTGGCGGCGAGGACTTCGACAACCGCCTCGTCACGTTCTTCACCGAGGAGTTCAAGCGCAAGAACAAGGGT	180
FN395032_L. infa... hsp70_ Portugal	ACGAACGGCGACACGCACCTTGGCGGCGAGGACTTCGACAACCGCCTCGTCACGTTCTTCACCGAGGAGTTCAAGCGCAAGAACAAGGGT	180
L. Tropica_hsp70	ACGAACGGCGACACACACCTTGGCGGCGAGGACTTCGACAACCGCCTCGTCACGTTCTTCACCGAGGAGTTCAAGCGCAAGAACAAGGGT	180
LN907846_L. Tropica_hsp70_Israel	ACGAACGGCGACACACACCTTGGCGGCGAGGACTTCGACAACCGCCTCGTCACGTTCTTCACCGAGGAGTTCAAGCGCAAGAACAAGGGT	180
MW658424_L. Tro...70_isolate_L192	ACGAACGGCGACACACACCTTGGCGGCGAGGACTTCGACAACCGCCTCGTCACGTTCTTCACCGAGGAGTTCAAGCGCAAGAACAAGGGT	180
MW658401_L. Tro...p70_isolate_PL5	ACGAACGGCGACACACACCTTGGCGGCGAGGACTTCGACAACCGCCTCGTCACGTTCTTCACCGAGGAGTTCAAGCGCAAGAACAAGGGT	180
MZ362273_L. Tro...p70_Afghanistan	ACGAACGGCGACACACACCTTGGCGGCGAGGACTTCGACAACCGCCTCGTCACGTTCTTCACCGAGGAGTTCAAGCGCAAGAACAAGGGT	180
FN395023_L. major_ hsp70_Spain	ACGAACGGCGATACACACCTTGGCGGCGAGGACTTCGACAACCGCCTCGTCACGTTCTTCACCGAGGAGTTCAAGCGCAAGAACAAGGGT	180
FN395022_ L. major_ hsp70_Sudan	ACGAACGGCGATACACACCTTGGCGGCGAGGACTTCGACAACCGCCTCGTCACGTTCTTCACCGAGGAGTTCAAGCGCAAGAACAAGGGT	180

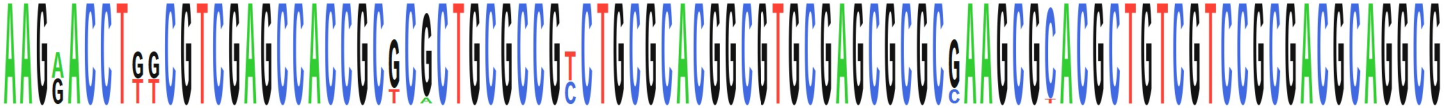

**Consensus**	AAGAACCTGGCGTCGAGCCACCGCGCGCTGCGCCGTCTGCGCACGGCGTGCGAGCGCGCGAAGCGCACGCTGTCGTCCGCGACGCAGGCG	
L. Panamensis_hsp70	AAGGACCTTTCGTCGAGCCACCGCGCGCTGCGCCGCCTGCGCACGGNGTGCGAGCGCGCGAAGCGCACGCTGTCGTCCGCGACGCAGGCG	270
EU599094_L. Pan...is_hsp70_Panama	AAGGACCTTTCGTCGAGCCACCGCGCGCTGCGCCGCCTGCGCACGGCGTGCGAGCGCGCGAAGCGCACGCTGTCGTCCGCGACGCAGGCG	270
FN395055_L. Pan...is_hsp70_Panama	AAGGACCTTTCGTCGAGCCACCGCGCGCTGCGCCGCCTGCGCACGGCGTGCGAGCGCGCGAAGCGCACGCTGTCGTCCGCGACGCAGGCG	270
KX573937_L. Pan...is_hsp70_Panama	AAGGACCTTTCGTCGAGCCACCGCGCGCTGCGCCGCCTGCGCACGGCGTGCGAGCGCGCGAAGCGCACGCTGTCGTCCGCGACGCAGGCG	270
KX573939_L. Pan...is_hsp70_Panama	AAGGACCTTTCGTCGAGCCACCGCGCGCTGCGCCGCCTGCGCACGGCGTGCGAGCGCGCGAAGCGCACGCTGTCGTCCGCGACGCAGGCG	270
KX573942_L. Pan...is_hsp70_Panama	AAGGACCTTTCGTCGAGCCACCGCGCGCTGCGCCGCCTGCGCACGGCGTGCGAGCGCGCGAAGCGCACGCTGTCGTCCGCGACGCAGGCG	270
KX573971_L. Pan...is_hsp70_Panama	AAGGACCTTTCGTCGAGCCACCGCGCGCTGCGCCGCCTGCGCACGGCGTGCGAGCGCGCGAAGCGCACGCTGTCGTCCGCGACGCAGGCG	270
KX573975_L. Pan...is_hsp70_Panama	AAGGACCTTTCGTCGAGCCACCGCGCGCTGCGCCGCCTGCGCACGGCGTGCGAGCGCGCGAAGCGCACGCTGTCGTCCGCGACGCAGGCG	270
OQ408896_L. Pan...is_hsp70_Panama	AAGGACCTTTCGTCGAGCCACCGCGCGCTGCGCCGCCTGCGCACGGCGTGCGAGCGCGCGAAGCGCACGCTGTCGTCCGCGACGCAGGCG	270
MW480568_L. Pa...s_hsp70_Norway	AAGGACCTTTCGTCGAGCCACCGCGCGCTGCGCCGCCTGCGCACGGCGTGCGAGCGCGCGAAGCGCACGCTGTCGTCCGCGACGCAGGCG	270
HF586406_L. guy...0_French Guiana	AAGGACCTTTCGTCGAGCCACCGCGCGCTGCGCCGCCTGCGCACGGCGTGCGAGCGCGCGAAGCGCACGCTGTCGTCCGCGACGCAGGCG	270
KX573934_L. guy...is_hsp70_Panama	AAGGACCTTTCGTCGAGCCACCGCGCGCTGCGCCGCCTGCGCACGGCGTGCGAGCGCGCGAAGCGCACGCTGTCGTCCGCGACGCAGGCG	270
KX574011_L. guy...is_hsp70_Panama	AAGGACCTTTCGTCGAGCCACCGCGCGCTGCGCCGCCTGCGCACGGCGTGCGAGCGCGCGAAGCGCACGCTGTCGTCCGCGACGCAGGCG	270
MW658405_ L. gu...70_isolate_PL11	AAGGACCTTTCGTCGAGCCACCGCGCGCTGCGCCGCCTGCGCACGGCGTGCGAGCGCGCGAAGCGCACGCTGTCGTCCGCGACGCAGGCG	270
MW658386_ L. gu...70_isolate_PL21	AAGGACCTTTCGTCGAGCCACCGCGCGCTGCGCCGCCTGCGCACGGCGTGCGAGCGCGCGAAGCGCACGCTGTCGTCCGCGACGCAGGCG	270
MW658403_ L. gu...p70_isolate_PL6	AAGGACCTTTCGTCGAGCCACCGCGCGCTGCGCCGCCTGCGCACGGCGTGCGAGCGCGCGAAGCGCACGCTGTCGTCCGCGACGCAGGCG	270
MG029123_L. ama...is_hsp70_Brazil	AAGAACCTGGCGTCGAGCCACCGCTCGCTGCGCCGTCTGCGCACGGCGTGCGAGCGCGCCAAGCGCACGCTGTCGTCCGCGACGCAGGCG	270
EU599090_L. amaz...sis_hsp70_Brazil	AAGAACCTGGCGTCGAGCCACCGCTCGCTGCGCCGTCTGCGCACGGCGTGCGAGCGCGCCAAGCGCACGCTGTCGTCCGCGACGCAGGCG	270
MF344859_L. ama...is_hsp70_Brazil	AAGAACCTGGCGTCGAGCCACCGCTCGCTGCGCCGTCTGCGCACGGCGTGCGAGCGCGCCAAGCGCACGCTGTCGTCCGCGACGCAGGCG	270
MF344857_L. ama...is_hsp70_Brazil	AAGAACCTGGCGTCGAGCCACCGCTCGCTGCGCCGTCTGCGCACGGCGTGCGAGCGCGCCAAGCGCACGCTGTCGTCCGCGACGCAGGCG	270
OP561801_L. amaz...is_hsp70_Bolivia	AAGAACCTGGCGTCGAGCCACCGCTCGCTGCGCCGTCTGCGCACGGCGTGCGAGCGCGCCAAGCGCACGCTGTCGTCCGCGACGCAGGCG	270
LN907831_L. ama...0_French Guiana	AAGAACCTGGCGTCGAGCCACCGCTCGCTGCGCCGTCTGCGCACGGCGTGCGAGCGCGCCAAGCGCACGCTGTCGTCCGCGACGCAGGCG	270
FN395039_L. braz...is_hsp70_Bolivia	AAGGACCTTTCGTCGAGCCACCGCGCGCTGCGCCGCCTGCGCACGGCGTGCGAGCGCGCGAAGCGCACGCTGTCGTCCGCGACGCAGGCG	270
FN395041_L. braz...is_hsp70_Bolivia	AAGGACCTTTCGTCGAGCCACCGCGCGCTGCGCCGCCTGCGCACGGCGTGCGAGCGCGCGAAGCGCACGCTGTCGTCCGCGACGCAGGCG	270
FR872760_L. braz...is_hsp70_Bolivia	AAGGACCTTTCGTCGAGCCACCGCGCACTGCGCCGTCTGCGCACGGCGTGCGAGCGCGCGAAGCGCACGCTGTCGTCCGCGACGCAGGCG	270
KX573945_L. bra...is_hsp70_Panama	AAGGACCTTTCGTCGAGCCACCGCGCACTGCGCCGTCTGCGCACGGCGTGCGAGCGCGCGAAGCGCACGCTGTCGTCCGCGACGCAGGCG	270
FR872763_L. bra...nsis_hsp70_Peru	AAGGACCTTTCGTCGAGCCACCGCGCACTGCGCCGTCTGCGCACGGCGTGCGAGCGCGCGAAGCGCACGCTGTCGTCCGCGACGCAGGCG	270
HF586401_L. mex...na_hsp70_Mexico	AAGAACCTGGCGTCGAGCCACCGCTCGCTGCGCCGTCTGCGCACGGCGTGCGAGCGCGCCAAGCGTACGCTGTCGTCCGCGACGCAGGCG	270
HF586413_L. mex...a_hsp70_Ecuador	AAGAACCTGGCGTCGAGCCACCGCTCGCTGCGCCGTCTGCGCACGGCGTGCGAGCGCGCCAAGCGCACGCTGTCGTCCGCGACGCAGGCG	270
FN395038_ L. mexicana_hsp70_Peru	AAGAACCTGGCGTCGAGCCACCGCTCGCTGCGCCGTCTGCGCACGGCGTGCGAGCGCGCCAAGCGCACGCTGTCGTCCGCGACGCAGGCG	270
EU599091_ L. me...a_hsp70_ Belize	AAGAACCTGGCGTCGAGCCACCGCTCGCTGCGCCGTCTGCGCACGGCGTGCGAGCGCGCCAAGCGTACGCTGTCGTCCGCGACGCAGGCG	270
MN728741_L. don...i_ hsp70_ India	AAGAACCTGGCGTCGAGCCACCGCGCGCTGCGCCGTCTGCGCACGGCGTGCGAGCGCGCGAAGCGCACGCTGTCGTCCGCGACGCAGGCG	270
MN728759_L. don...i_ hsp70_ Sudan	AAGAACCTGGCGTCGAGCCACCGCGCGCTGCGCCGTCTGCGCACGGCGTGCGAGCGCGCGAAGCGCACGCTGTCGTCCGCGACGCAGGCG	270
MN728753_L. inf...m_ hsp70_ Malta	AAGAACCTGGCGTCGAGCCACCGCGCGCTGCGCCGTCTGCGCACGGCGTGCGAGCGCGCGAAGCGCACGCTGTCGTCCGCGACGCAGGCG	270
MN728756_L. inf...m_ hsp70_ Sudan	AAGAACCTGGCGTCGAGCCACCGCGCGCTGCGCCGTCTGCGCACGGCGTGCGAGCGCGCGAAGCGCACGCTGTCGTCCGCGACGCAGGCG	270
FN395032_L. infa... hsp70_ Portugal	AAGAACCTGGCGTCGAGCCACCGCGCGCTGCGCCGTCTGCGCACGGCGTGCGAGCGCGCGAAGCGCACGCTGTCGTCCGCGACGCAGGCG	270
L. Tropica_hsp70	AAGAACCTGGCGTCGAGCCACCGCGCGCTGCGCCGTCTGCGCACGGCGTGCGAGCGCGCGAAGCGCACGCTGTCGTCCGCGACGCAGGCG	270
LN907846_L. Tropica_hsp70_Israel	AAGAACCTGGCGTCGAGCCACCGCGCGCTGCGCCGTCTGCGCACGGCGTGCGAGCGCGCGAAGCGCACGCTGTCGTCCGCGACGCAGGCG	270
MW658424_L. Tro...70_isolate_L192	AAGAACCTGGCGTCGAGCCACCGCGCGCTGCGCCGTCTGCGCACGGCGTGCGAGCGCGCGAAGCGCACGCTGTCGTCCGCGACGCAGGCG	270
MW658401_L. Tro...p70_isolate_PL5	AAGAACCTGGCGTCGAGCCACCGCGCGCTGCGCCGTCTGCGCACGGCGTGCGAGCGCGCGAAGCGCACGCTGTCGTCCGCGACGCAGGCG	270
MZ362273_L. Tro...p70_Afghanistan	AAGAACCTGGCGTCGAGCCACCGCGCGCTGCGCCGTCTGCGCACGGCGTGCGAGCGCGCGAAGCGCACGCTGTCGTCCGCGACGCAGGCG	270
FN395023_L. major_ hsp70_Spain	AAGAACCTGGCGTCGAGCCACCGCGCGCTGCGCCGTCTGCGCACGGCGTGCGAGCGCGCGAAGCGCACGCTGTCGTCCGCGACGCAGGCG	270
FN395022_ L. major_ hsp70_Sudan	AAGAACCTGGCGTCGAGCCACCGCGCGCTGCGCCGTCTGCGCACGGCGTGCGAGCGCGCGAAGCGCACGCTGTCGTCCGCGACGCAGGCG	270

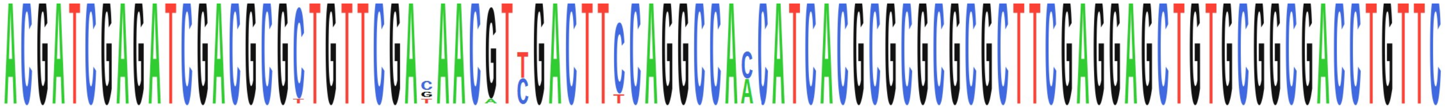

**Consensus**	ACGATCGAGATCGACGCGCTGTTCGANAACGTTGACTTCCAGGCCACCATCACGCGCGCGCGCTTCGAGGAGCTGTGCGGCGACCTGTTC	
L. Panamensis_hsp70	ACGATCGAGATCGACGCGCTGTTCGACAACGTCGACTTCCAGGCCAACATCACGCGCGCGCGCTTCGAGGAGCTGTGCGGCGACCTGTTC	360
EU599094_L. Pan...is_hsp70_Panama	ACGATCGAGATCGACGCGCTGTTCGACAACGTCGACTTCCAGGCCAACATCACGCGCGCGCGCTTCGAGGAGCTGTGCGGCGACCTGTTC	360
FN395055_L. Pan...is_hsp70_Panama	ACGATCGAGATCGACGCGCTGTTCGACAACGTCGACTTCCAGGCCAACATCACGCGCGCGCGCTTCGAGGAGCTGTGCGGCGACCTGTTC	360
KX573937_L. Pan...is_hsp70_Panama	ACGATCGAGATCGACGCGCTGTTCGACAACGTCGACTTCCAGGCCAACATCACGCGCGCGCGCTTCGAGGAGCTGTGCGGCGACCTGTTC	360
KX573939_L. Pan...is_hsp70_Panama	ACGATCGAGATCGACGCGCTGTTCGACAACGTCGACTTCCAGGCCAACATCACGCGCGCGCGCTTCGAGGAGCTGTGCGGCGACCTGTTC	360
KX573942_L. Pan...is_hsp70_Panama	ACGATCGAGATCGACGCGCTGTTCGACAACGTCGACTTCCAGGCCAACATCACGCGCGCGCGCTTCGAGGAGCTGTGCGGCGACCTGTTC	360
KX573971_L. Pan...is_hsp70_Panama	ACGATCGAGATCGACGCGCTGTTCGACAACGTCGACTTCCAGGCCAACATCACGCGCGCGCGCTTCGAGGAGCTGTGCGGCGACCTGTTC	360
KX573975_L. Pan...is_hsp70_Panama	ACGATCGAGATCGACGCGCTGTTCGACAACGTCGACTTCCAGGCCAACATCACGCGCGCGCGCTTCGAGGAGCTGTGCGGCGACCTGTTC	360
OQ408896_L. Pan...is_hsp70_Panama	ACGATCGAGATCGACGCGCTGTTCGACAACGTCGACTTCCAGGCCAACATCACGCGCGCGCGCTTCGAGGAGCTGTGCGGCGACCTGTTC	360
MW480568_L. Pa...s_hsp70_Norway	ACGATCGAGATCGACGCGCTGTTCGACAACGTCGACTTCCAGGCCAACATCACGCGCGCGCGCTTCGAGGAGCTGTGCGGCGACCTGTTC	360
HF586406_L. guy...0_French Guiana	ACGATCGAGATCGACGCGCTGTTCGACAACGTCGACTTCCAGGCCAACATCACGCGCGCGCGCTTCGAGGAGCTGTGCGGCGACCTGTTC	360
KX573934_L. guy...is_hsp70_Panama	ACGATCGAGATCGACGCGCTGTTCGACAACGTCGACTTCCAGGCCAACATCACGCGCGCGCGCTTCGAGGAGCTGTGCGGCGACCTGTTC	360
KX574011_L. guy...is_hsp70_Panama	ACGATCGAGATCGACGCGCTGTTCGACAACGTCGACTTCCAGGCCAACATCACGCGCGCGCGCTTCGAGGAGCTGTGCGGCGACCTGTTC	360
MW658405_ L. gu...70_isolate_PL11	ACGATCGAGATCGACGCGCTGTTCGACAACGTCGACTTCCAGGCCAACATCACGCGCGCGCGCTTCGAGGAGCTGTGCGGCGACCTGTTC	360
MW658386_ L. gu...70_isolate_PL21	ACGATCGAGATCGACGCGCTGTTCGACAACGTCGACTTCCAGGCCAACATCACGCGCGCGCGCTTCGAGGAGCTGTGCGGCGACCTGTTC	360
MW658403_ L. gu...p70_isolate_PL6	ACGATCGAGATCGACGCGCTGTTCGACAACGTCGACTTCCAGGCCAACATCACGCGCGCGCGCTTCGAGGAGCTGTGCGGCGACCTGTTC	360
MG029123_L. ama...is_hsp70_Brazil	ACGATCGAGATCGACGCGCTGTTCGATAACGTTGACTTCCAGGCYACCATCACGCGCGCGCGCTTCGAGGAGCTGTGCGGCGACCTGTTC	360
EU599090_L. amaz...sis_hsp70_Brazil	ACGATCGAGATCGACGCGCTGTTCGATAACGTTGACTTCCAGGCCACCATCACGCGCGCGCGCTTCGAGGAGCTGTGCGGCGACCTGTTC	360
MF344859_L. ama...is_hsp70_Brazil	ACGATCGAGATCGACGCGCTGTTCGATAACGTTGACTTCCAGGCCACCATCACGCGCGCGCGCTTCGAGGAGCTGTGCGGCGACCTGTTC	360
MF344857_L. ama...is_hsp70_Brazil	ACGATCGAGATCGACGCGCTGTTCGATAACGTTGACTTCCAGGCCACCATCACGCGCGCGCGCTTCGAGGAGCTGTGCGGCGACCTGTTC	360
OP561801_L. amaz...is_hsp70_Bolivia	ACGATCGAGATCGACGCGCTGTTCGATAACGTTGACTTCCAGGCCACCATCACGCGCGCGCGCTTCGAGGAGCTGTGCGGCGACCTGTTC	360
LN907831_L. ama...0_French Guiana	ACGATCGAGATCGACGCGCTGTTCGATAACGTTGACTTCCAGGCCACCATCACGCGCGCGCGCTTCGAGGAGCTGTGCGGCGACCTGTTC	360
FN395039_L. braz...is_hsp70_Bolivia	ACGATCGAGATCGACGCGCTGTTCGACAACGTCGACTTCCAGGCCAACATCACGCGCGCGCGCTTCGAGGAGCTGTGCGGCGACCTGTTC	360
FN395041_L. braz...is_hsp70_Bolivia	ACGATCGAGATCGACGCGCTGTTCGACAACGTCGACTTCCAGGCCAACATCACGCGCGCGCGCTTCGAGGAGCTGTGCGGCGACCTGTTC	360
FR872760_L. braz...is_hsp70_Bolivia	ACGATCGAGATCGACGCGCTGTTCGACAACGTCGACTTCCAGGCCAACATCACGCGCGCGCGCTTCGAGGAGCTGTGCGGCGACCTGTTC	360
KX573945_L. bra...is_hsp70_Panama	ACGATCGAGATCGACGCGCTGTTCGACAACGTCGACTTCCAGGCCAACATCACGCGCGCGCGCTTCGAGGAGCTGTGCGGCGACCTGTTC	360
FR872763_L. bra...nsis_hsp70_Peru	ACGATCGAGATCGACGCGCTGTTCGACAACGTCGACTTCCAGGCCAACATCACGCGCGCGCGCTTCGAGGAGCTGTGCGGCGACCTGTTC	360
HF586401_L. mex...na_hsp70_Mexico	ACGATCGAGATCGACGCGCTGTTCGATAACGTTGACTTCCAGGCCACCATCACGCGCGCGCGCTTCGAGGAGCTGTGCGGCGACCTGTTC	360
HF586413_L. mex...a_hsp70_Ecuador	ACGATCGAGATCGACGCGCTGTTCGATAACGTTGACTTCCAGGCCACCATCACGCGCGCGCGCTTCGAGGAGCTGTGCGGCGACCTGTTC	360
FN395038_ L. mexicana_hsp70_Peru	ACGATCGAGATCGACGCGCTGTTCGATAACGTTGACTTCCAGGCCACCATCACGCGCGCGCGCTTCGAGGAGCTGTGCGGCGACCTGTTC	360
EU599091_ L. me...a_hsp70_ Belize	ACGATCGAGATCGACGCGCTGTTCGATAACGTTGACTTCCAGGCCACCATCACGCGCGCGCGCTTCGAGGAGCTGTGCGGCGACCTGTTC	360
MN728741_L. don...i_ hsp70_ India	ACGATCGAGATCGACGCGCTGTTCGAGAACGTTGACTTTCAGGCCACCATCACGCGCGCGCGCTTCGAGGAGCTGTGCGGCGACCTGTTC	360
MN728759_L. don...i_ hsp70_ Sudan	ACGATCGAGATCGACGCGCTGTTCGAGAACGTTGACTTTCAGGCCACCATCACGCGCGCGCGCTTCGAGGAGCTGTGCGGCGACCTGTTC	360
MN728753_L. inf...m_ hsp70_ Malta	ACGATCGAGATCGACGCGTTGTTCGAGAACGTTGACTTTCAGGCCACCATCACGCGCGCGCGCTTCGAGGAGCTGTGCGGCGACCTGTTC	360
MN728756_L. inf...m_ hsp70_ Sudan	ACGATCGAGATCGACGCGCTGTTCGAGAACGTTGACTTTCAGGCCACCATCACGCGCGCGCGCTTCGAGGAGCTGTGCGGCGACCTGTTC	360
FN395032_L. infa... hsp70_ Portugal	ACGATCGAGATCGACGCGTTGTTCGAGAACGTTGACTTTCAGGCCACCATCACGCGCGCGCGCTTCGAGGAGCTGTGCGGCGACCTGTTC	360
L. Tropica_hsp70	ACGATCGAGATCGACGCGCTGTTCGAGAACGTTGACTTCCAGGCCACCATCACGCGCGCGCGCTTCGAGGAGCTGTGCGGCGACCTGTTC	360
LN907846_L. Tropica_hsp70_Israel	ACGATCGAGATCGACGCGCTGTTCGAGAACGTTGACTTCCAGGCCACCATCACGCGCGCGCGCTTCGAGGAGCTGTGCGGCGACCTGTTC	360
MW658424_L. Tro...70_isolate_L192	ACGATCGAGATCGACGCGCTGTTCGAGAACGTTGACTTCCAGGCCACCATCACGCGCGCGCGCTTCGAGGAGCTGTGCGGCGACCTGTTC	360
MW658401_L. Tro...p70_isolate_PL5	ACGATCGAGATCGACGCGCTGTTCGAGAACGTTGACTTCCAGGCCACCATCACGCGCGCGCGCTTCGAGGAGCTGTGCGGCGACCTGTTC	360
MZ362273_L. Tro...p70_Afghanistan	ACGATCGAGATCGACGCGCTGTTCGAGAACGTTGACTTCCAGGCCACCATCACGCGCGCGCGCTTCGAGGAGCTGTGCGGCGACCTGTTC	360
FN395023_L. major_ hsp70_Spain	ACGATCGAGATCGACGCGCTGTTCGAGAACATTGACTTCCAGGCCACCATCACGCGCGCGCGCTTCGAGGAGCTGTGCGGCGACCTGTTC	360
FN395022_ L. major_ hsp70_Sudan	ACGATCGAGATCGACGCGCTGTTCGAGAACATTGACTTCCAGGCCACCATCACGCGCGCGCGCTTCGAGGAGCTGTGCGGCGACCTGTTC	360

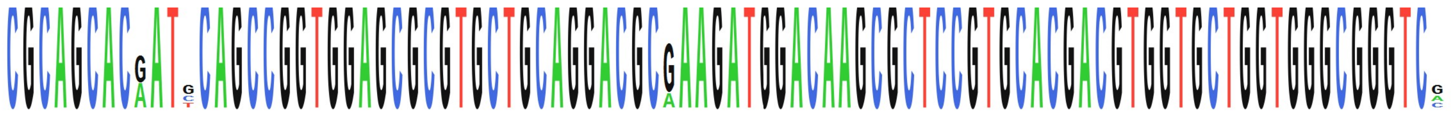

**Consensus**	CGCAGCACGATNCAGCCGGTGGAGCGCGTGCTGCAGGACGCGAAGATGGACAAGCGCTCCGTGCACGACGTGGTGCTGGTGGGCGGTCN	
L. Panamensis_hsp70	CGCAGCACAATGCAGCCGGTGGAGCGCGTGCTGCAGGACGCGAAGATGGACAAGCGCTCCGTGCACGACGTGGTGCTGGTGGGCGGTCG	450
EU599094_L. Pan...is_hsp70_Panama	CGCAGCACAATGCAGCCGGTGGAGCGCGTGCTGCAGGACGCGAAGATGGACAAGCGCTCCGTGCACGACGTGGTGCTGGTGGGCGGTCG	450
FN395055_L. Pan...is_hsp70_Panama	CGCAGCACAATGCAGCCGGTGGAGCGCGTGCTGCAGGACGCGAAGATGGACAAGCGCTCCGTGCACGACGTGGTGCTGGTGGGCGGTCG	450
KX573937_L. Pan...is_hsp70_Panama	CGCAGCACAATGCAGCCGGTGGAGCGCGTGCTGCAGGACGCGAAGATGGACAAGCGCTCCGTGCACGACGTGGTGCTGGTGGGCGGTCG	450
KX573939_L. Pan...is_hsp70_Panama	CGCAGCACAATGCAGCCGGTGGAGCGCGTGCTGCAGGACGCGAAGATGGACAAGCGCTCCGTGCACGACGTGGTGCTGGTGGGCGGTCG	450
KX573942_L. Pan...is_hsp70_Panama	CGCAGCACAATGCAGCCGGTGGAGCGCGTGCTGCAGGACGCGAAGATGGACAAGCGCTCCGTGCACGACGTGGTGCTGGTGGGCGGTCG	450
KX573971_L. Pan...is_hsp70_Panama	CGCAGCACAATGCAGCCGGTGGAGCGCGTGCTGCAGGACGCGAAGATGGACAAGCGCTCCGTGCACGACGTGGTGCTGGTGGGCGGTCG	450
KX573975_L. Pan...is_hsp70_Panama	CGCAGCACAATGCAGCCGGTGGAGCGCGTGCTGCAGGACGCGAAGATGGACAAGCGCTCCGTGCACGACGTGGTGCTGGTGGGCGGTCG	450
OQ408896_L. Pan...is_hsp70_Panama	CGCAGCACAATGCAGCCGGTGGAGCGCGTGCTGCAGGACGCGAAGATGGACAAGCGCTCCGTGCACGACGTGGTGCTGGTGGGCGGTCG	450
MW480568_L. Pa...s_hsp70_Norway	CGCAGCACAATGCAGCCGGTGGAGCGCGTGCTGCAGGACGCGAAGATGGACAAGCGCTCCGTGCACGACGTGGTGCTGGTGGGCGGTCG	450
HF586406_L. guy...0_French Guiana	CGCAGCACAATGCAGCCGGTGGAGCGCGTGCTGCAGGACGCGAAGATGGACAAGCGCTCCGTGCACGACGTGGTGCTGGTGGGCGGTCG	450
KX573934_L. guy...is_hsp70_Panama	CGCAGCACAATGCAGCCGGTGGAGCGCGTGCTGCAGGACGCGAAGATGGACAAGCGCTCCGTGCACGACGTGGTGCTGGTGGGCGGTCG	450
KX574011_L. guy...is_hsp70_Panama	CGCAGCACAATGCAGCCGGTGGAGCGCGTGCTGCAGGACGCGAAGATGGACAAGCGCTCCGTGCACGACGTGGTGCTGGTGGGCGGTCG	450
MW658405_ L. gu...70_isolate_PL11	CGCAGCACAATGCAGCCGGTGGAGCGCGTGCTGCAGGACGCGAAGATGGACAAGCGCTCCGTGCACGACGTGGTGCTGGTGGGCGGTCG	450
MW658386_ L. gu...70_isolate_PL21	CGCAGCACAATGCAGCCGGTGGAGCGCGTGCTGCAGGACGCGAAGATGGACAAGCGCTCCGTGCACGACGTGGTGCTGGTGGGCGGTCG	450
MW658403_ L. gu...p70_isolate_PL6	CGCAGCACAATGCAGCCGGTGGAGCGCGTGCTGCAGGACGCGAAGATGGACAAGCGCTCCGTGCACGACGTGGTGCTGGTGGGCGGTCG	450
MG029123_L. ama...is_hsp70_Brazil	CGCAGCACGATTCAGCCGGTGGAGCGCGTGCTGCAGGACGCAAAGATGGACAAGCGCTCCGTGCACGACGTGGTGCTGGTGGGCGGTCC	450
EU599090_L. amaz...sis_hsp70_Brazil	CGCAGCACGATTCAGCCGGTGGAGCGCGTGCTGCAGGACGCAAAGATGGACAAGCGCTCCGTGCACGACGTGGTGCTGGTGGGCGGTCC	450
MF344859_L. ama...is_hsp70_Brazil	CGCAGCACGATTCAGCCGGTGGAGCGCGTGCTGCAGGACGCAAAGATGGACAAGCGCTCCGTGCACGACGTGGTGCTGGTGGGCGGTCC	450
MF344857_L. ama...is_hsp70_Brazil	CGCAGCACGATTCAGCCGGTGGAGCGCGTGCTGCAGGACGCAAAGATGGACAAGCGCTCCGTGCACGACGTGGTGCTGGTGGGCGGTCC	450
OP561801_L. amaz...is_hsp70_Bolivia	CGCAGCACGATTCAGCCGGTGGAGCGCGTGCTGCAGGACGCAAAGATGGACAAGCGCTCCGTGCACGACGTGGTGCTGGTGGGCGGTCC	450
LN907831_L. ama...0_French Guiana	CGCAGCACGATTCAGCCGGTGGAGCGCGTGCTGCAGGACGCAAAGATGGACAAGCGCTCCGTGCACGACGTGGTGCTGGTGGGCGGTCC	450
FN395039_L. braz...is_hsp70_Bolivia	CGCAGCACAATGCAGCCGGTGGAGCGCGTGCTGCAGGACGCGAAGATGGACAAGCGCTCCGTGCACGACGTGGTGCTGGTGGGCGGTCG	450
FN395041_L. braz...is_hsp70_Bolivia	CGCAGCACAATGCAGCCGGTGGAGCGCGTGCTGCAGGACGCGAAGATGGACAAGCGCTCCGTGCACGACGTGGTGCTGGTGGGCGGTCG	450
FR872760_L. braz...is_hsp70_Bolivia	CGCAGCACAATGCAGCCGGTGGAGCGCGTGCTGCAGGACGCGAAGATGGACAAGCGCTCCGTGCACGACGTGGTGCTGGTGGGCGGTCG	450
KX573945_L. bra...is_hsp70_Panama	CGCAGCACAATGCAGCCGGTGGAGCGCGTGCTGCAGGACGCGAAGATGGACAAGCGCTCCGTGCACGACGTGGTGCTGGTGGGCGGTCG	450
FR872763_L. bra...nsis_hsp70_Peru	CGCAGCACAATGCAGCCGGTGGAGCGCGTGCTGCAGGACGCGAAGATGGACAAGCGCTCCGTGCACGACGTGGTGCTGGTGGGCGGTCG	450
HF586401_L. mex...na_hsp70_Mexico	CGCAGCACGATTCAGCCGGTGGAGCGCGTGCTGCAGGACGCAAAGATGGACAAGCGCTCCGTGCACGACGTGGTGCTGGTGGGCGGTCC	450
HF586413_L. mex...a_hsp70_Ecuador	CGCAGCACGATTCAGCCGGTGGAGCGCGTGCTGCAGGACGCAAAGATGGACAAGCGCTCCGTGCACGACGTGGTGCTGGTGGGCGGTCC	450
FN395038_ L. mexicana_hsp70_Peru	CGCAGCACGATTCAGCCGGTGGAGCGCGTGCTGCAGGACGCAAAGATGGACAAGCGCTCCGTGCACGACGTGGTGCTGGTGGGCGGTCC	450
EU599091_ L. me...a_hsp70_ Belize	CGCAGCACGATTCAGCCGGTGGAGCGCGTGCTGCAGGACGCAAAGATGGACAAGCGCTCCGTGCACGACGTGGTGCTGGTGGGCGGTCC	450
MN728741_L. don...i_ hsp70_ India	CGCAGCACGATCCAGCCGGTGGAGCGCGTGCTGCAGGACGCGAAGATGGACAAGCGCTCCGTGCACGACGTGGTGCTGGTGGGCGGTCA	450
MN728759_L. don...i_ hsp70_ Sudan	CGCAGCACGATCCAGCCGGTGGAGCGCGTGCTGCAGGACGCGAAGATGGACAAGCGCTCCGTGCACGACGTGGTGCTGGTGGGCGGTCA	450
MN728753_L. inf...m_ hsp70_ Malta	CGCAGCACGATCCAGCCGGTGGAGCGCGTGCTGCAGGACGCGAAGATGGACAAGCGCTCCGTGCACGACGTGGTGCTGGTGGGCGGTCA	450
MN728756_L. inf...m_ hsp70_ Sudan	CGCAGCACGATCCAGCCGGTGGAGCGCGTGCTGCAGGACGCGAAGATGGACAAGCGCTCCGTGCACGACGTGGTGCTGGTGGGCGGTCA	450
FN395032_L. infa... hsp70_ Portugal	CGCAGCACGATCCAGCCGGTGGAGCGCGTGCTGCAGGACGCGAAGATGGACAAGCGCTCCGTGCACGACGTGGTGCTGGTGGGCGGTCA	450
L. Tropica_hsp70	CGCAGCACGATCCAGCCGGTGGAGCGCGTGCTGCAGGACGCGAAGATGGACAAGCGCTCCGTGCACGACGTGGTGCTGGTGGGCGGTCA	450
LN907846_L. Tropica_hsp70_Israel	CGCAGCACGATCCAGCCGGTGGAGCGCGTGCTGCAGGACGCGAAGATGGACAAGCGCTCCGTGCACGACGTGGTGCTGGTGGGCGGTCA	450
MW658424_L. Tro...70_isolate_L192	CGCAGCACGATCCAGCCGGTGGAGCGCGTGCTGCAGGACGCGAAGATGGACAAGCGCTCCGTGCACGACGTGGTGCTGGTGGGCGGTCA	450
MW658401_L. Tro...p70_isolate_PL5	CGCAGCACGATCCAGCCGGTGGAGCGCGTGCTGCAGGACGCGAAGATGGACAAGCGCTCCGTGCACGACGTGGTGCTGGTGGGCGGTCA	450
MZ362273_L. Tro...p70_Afghanistan	CGCAGCACGATCCAGCCGGTGGAGCGCGTGCTGCAGGACGCGAAGATGGACAAGCGCTCCGTGCACGACGTGGTGCTGGTGGGCGGTCA	450
FN395023_L. major_ hsp70_Spain	CGCAGCACGATCCAGCCGGTGGAGCGCGTGCTGCAGGACGCGAAGATGGACAAGCGCTCCGTGCACGACGTGGTGCTGGTGGGCGGTCA	450
FN395022_ L. major_ hsp70_Sudan	CGCAGCACGATCCAGCCGGTGGAGCGCGTGCTGCAGGACGCGAAGATGGACAAGCGCTCCGTGCACGACGTGGTGCTGGTGGGCGGTCA	450

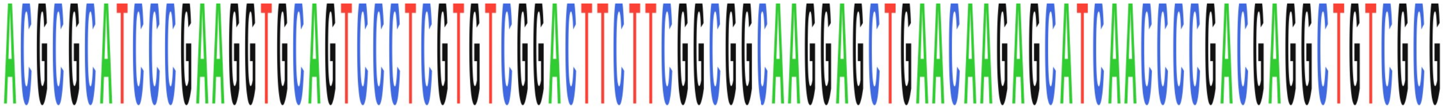

Consensus	ACGCGCATCCCGAAGGTGCAGTCCCTCGTGTCGGACTTCTTCGGCGGCAAGGAGCTGAACAAGAGCATCAACCCCGACGAGGCTGTCGCG	
L. Panamensis_hsp70	ACGCGCATCCCGAAGGTGCAGTCCCTCGTGTCGGACTTCTTCGGCGGCAAGGAGCTGAACAAGAGCATCAACCCCGACGAGGCTGTCGCG	540
EU599094_L. Pan…is_hsp70_Panama	ACGCGCATCCCGAAGGTGCAGTCCCTCGTGTCGGACTTCTTCGGCGGCAAGGAGCTGAACAAGAGCATCAACCCCGACGAGGCTGTCGCG	540
FN395055_L. Pan…is_hsp70_Panama	ACGCGCATCCCGAAGGTGCAGTCCCTCGTGTCGGACTTCTTCGGCGGCAAGGAGCTGAACAAGAGCATCAACCCCGACGAGGCTGTCGCG	540
KX573937_L. Pan…is_hsp70_Panama	ACGCGCATCCCGAAGGTGCAGTCCCTCGTGTCGGACTTCTTCGGCGGCAAGGAGCTGAACAAGAGCATCAACCCCGACGAGGCTGTCGCG	540
KX573939_L. Pan…is_hsp70_Panama	ACGCGCATCCCGAAGGTGCAGTCCCTCGTGTCGGACTTCTTCGGCGGCAAGGAGCTGAACAAGAGCATCAACCCCGACGAGGCTGTCGCG	540
KX573942_L. Pan…is_hsp70_Panama	ACGCGCATCCCGAAGGTGCAGTCCCTCGTGTCGGACTTCTTCGGCGGCAAGGAGCTGAACAAGAGCATCAACCCCGACGAGGCTGTCGCG	540
KX573971_L. Pan…is_hsp70_Panama	ACGCGCATCCCGAAGGTGCAGTCCCTCGTGTCGGACTTCTTCGGCGGCAAGGAGCTGAACAAGAGCATCAACCCCGACGAGGCTGTCGCG	540
KX573975_L. Pan…is_hsp70_Panama	ACGCGCATCCCGAAGGTGCAGTCCCTCGTGTCGGACTTCTTCGGCGGCAAGGAGCTGAACAAGAGCATCAACCCCGACGAGGCTGTCGCG	540
OQ408896_L. Pan…is_hsp70_Panama	ACGCGCATCCCGAAGGTGCAGTCCCTCGTGTCGGACTTCTTCGGCGGCAAGGAGCTGAACAAGAGCATCAACCCCGACGAGGCTGTCGCG	540
MW480568_L. Pa…s_hsp70_Norway	ACGCGCATCCCGAAGGTGCAGTCCCTCGTGTCGGACTTCTTCGGCGGCAAGGAGCTGAACAAGAGCATCAACCCCGACGAGGCTGTCGCG	540
HF586406_L. guy…0_French Guiana	ACGCGCATCCCGAAGGTGCAGTCCCTCGTGTCGGACTTCTTCGGCGGCAAGGAGCTGAACAAGAGCATCAACCCCGACGAGGCTGTCGCG	540
KX573934_L. guy…is_hsp70_Panama	ACGCGCATCCCGAAGGTGCAGTCCCTCGTGTCGGACTTCTTCGGCGGCAAGGAGCTGAACAAGAGCATCAACCCCGACGAGGCTGTCGCG	540
KX574011_L. guy…is_hsp70_Panama	ACGCGCATCCCGAAGGTGCAGTCCCTCGTGTCGGACTTCTTCGGCGGCAAGGAGCTGAACAAGAGCATCAACCCCGACGAGGCTGTCGCG	540
MW658405_ L. gu…70_isolate_PL11	ACGCGCATCCCGAAGGTGCAGTCCCTCGTGTCGGACTTCTTCGGCGGCAAGGAGCTGAACAAGAGCATCAACCCCGACGAGGCTGTCGCG	540
MW658386_ L. gu…70_isolate_PL21	ACGCGCATCCCGAAGGTGCAGTCCCTCGTGTCGGACTTCTTCGGCGGCAAGGAGCTGAACAAGAGCATCAACCCCGACGAGGCTGTCGCG	540
MW658403_ L. gu…p70_isolate_PL6	ACGCGCATCCCGAAGGTGCAGTCCCTCGTGTCGGACTTCTTCGGCGGCAAGGAGCTGAACAAGAGCATCAACCCCGACGAGGCTGTCGCG	540
MG029123_L. ama…is_hsp70_Brazil	ACGCGCATCCCGAAGGTGCAGTCCCTCGTGTCGGACTTCTTCGGCGGCAAGGAGCTGAACAAGAGCATCAACCCCGACGAGGCTGTCGCG	540
EU599090_L. amaz…sis_hsp70_Brazil	ACGCGCATCCCGAAGGTGCAGTCCCTCGTGTCGGACTTCTTCGGCGGCAAGGAGCTGAACAAGAGCATCAACCCCGACGAGGCTGTCGCG	540
MF344859_L. ama…is_hsp70_Brazil	ACGCGCATCCCGAAGGTGCAGTCCCTCGTGTCGGACTTCTTCGGCGGCAAGGAGCTGAACAAGAGCATCAACCCCGACGAGGCTGTCGCG	540
MF344857_L. ama…is_hsp70_Brazil	ACGCGCATCCCGAAGGTGCAGTCCCTCGTGTCGGACTTCTTCGGCGGCAAGGAGCTGAACAAGAGCATCAACCCCGACGAGGCTGTCGCG	540
OP561801_L. amaz…is_hsp70_Bolivia	ACGCGCATCCCGAAGGTGCAGTCCCTCGTGTCGGACTTCTTCGGCGGCAAGGAGCTGAACAAGAGCATCAACCCCGACGAGGCTGTCGCG	540
LN907831_L. ama…0_French Guiana	ACGCGCATCCCGAAGGTGCAGTCCCTCGTGTCGGACTTCTTCGGCGGCAAGGAGCTGAACAAGAGCATCAACCCCGACGAGGCTGTCGCG	540
FN395039_L. braz…is_hsp70_Bolivia	ACGCGCATCCCGAAGGTGCAGTCCCTCGTGTCGGACTTCTTCGGCGGCAAGGAGCTGAACAAGAGCATCAACCCCGACGAGGCTGTCGCG	540
FN395041_L. braz…is_hsp70_Bolivia	ACGCGCATCCCGAAGGTGCAGTCCCTCGTNTCGGACTTCTTCGGCGGCAAGGAGCTGAACAAGAGCATCAACCCCGACGAGGCTGTCGCG	540
FR872760_L. braz…is_hsp70_Bolivia	ACGCGCATCCCGAAGGTGCAGTCCCTCGTGTCGGACTTCTTCGGCGGCAAGGAGCTGAACAAGAGCATCAACCCCGACGAGGCTGTCGCG	540
KX573945_L. bra…is_hsp70_Panama	ACGCGCATCCCGAAGGTGCAGTCCCTCGTGTCGGACTTCTTCGGCGGCAAGGAGCTGAACAAGAGCATCAACCCCGACGAGGCTGTCGCG	540
FR872763_L. bra…nsis_hsp70_Peru	ACGCGCATCCCGAAGGTGCAGTCCCTCGTGTCGGACTTCTTCGGCGGCAAGGAGCTGAACAAGAGCATCAACCCCGACGAGGCTGTCGCG	540
HF586401_L. mex…na_hsp70_Mexico	ACGCGCATCCCGAAGGTGCAGTCCCTCGTGTCGGACTTCTTCGGCGGCAAGGAGCTGAACAAGAGCATCAACCCCGACGAGGCTGTCGCG	540
HF586413_L. mex…a_hsp70_Ecuador	ACGCGCATCCCGAAGGTGCAGTCCCTCGTGTCGGACTTCTTCGGCGGCAAGGAGCTGAACAAGAGCATCAACCCCGACGAGGCTGTCGCG	540
FN395038_ L. mexicana_hsp70_Peru	ACGCGCATCCCGAAGGTGCAGTCCCTCGTGTCGGACTTCTTCGGCGGCAAGGAGCTGAACAAGAGCATCAACCCCGACGAGGCTGTCGCG	540
EU599091_ L. me…a_hsp70_ Belize	ACGCGCATCCCGAAGGTGCAGTCCCTCGTGTCGGACTTCTTCGGCGGCAAGGAGCTGAACAAGAGCATCAACCCCGACGAGGCTGTCGCG	540
MN728741_L. don…i_ hsp70_ India	ACGCGCATCCCGAAGGTGCAGTCCCTCGTGTCGGACTTCTTCGGCGGCAAGGAGCTGAACAAGAGCATCAACCCCGACGAGGCTGTCGCG	540
MN728759_L. don…i_ hsp70_ Sudan	ACGCGCATCCCGAAGGTGCAGTCCCTCGTGTCGGACTTCTTCGGCGGCAAGGAGCTGAACAAGAGCATCAACCCCGACGAGGCTGTCGCG	540
MN728753_L. inf…m_ hsp70_ Malta	ACGCGCATCCCGAAGGTGCAGTCCCTCGTGTCGGACTTCTTCGGCGGCAAGGAGCTGAACAAGAGCATCAACCCCGACGAGGCTGTCGCG	540
MN728756_L. inf…m_ hsp70_ Sudan	ACGCGCATCCCGAAGGTGCAGTCCCTCGTGTCGGACTTCTTCGGCGGCAAGGAGCTGAACAAGAGCATCAACCCCGACGAGGCTGTCGCG	540
FN395032_L. infa… hsp70_ Portugal	ACGCGCATCCCGAAGGTGCAGTCCCTCGTGTCGGACTTCTTCGGCGGCAAGGAGCTGAACAAGAGCATCAACCCCGACGAGGCTGTCGCG	540
L. Tropica_hsp70	ACGCGCATCCCGAAGGTGCAGTCCCTCGTGTCGGACTTCTTCGGCGGCAAGGAGCTGAACAAGAGCATCAACCCCGACGAGGCTGTCGCG	540
LN907846_L. Tropica_hsp70_Israel	ACGCGCATCCCGAAGGTGCAGTCCCTCGTGTCGGACTTCTTCGGCGGCAAGGAGCTGAACAAGAGCATCAACCCCGACGAGGCTGTCGCG	540
MW658424_L. Tro…70_isolate_L192	ACGCGCATCCCGAAGGTGCAGTCCCTCGTGTCGGACTTCTTCGGCGGCAAGGAGCTGAACAAGAGCATCAACCCCGACGAGGCTGTCGCG	540
MW658401_L. Tro…p70_isolate_PL5	ACGCGCATCCCGAAGGTGCAGTCCCTCGTGTCGGACTTCTTCGGCGGCAAGGAGCTGAACAAGAGCATCAACCCCGACGAGGCTGTCGCG	540
MZ362273_L. Tro…p70_Afghanistan	ACGCGCATCCCGAAGGTGCAGTCCCTCGTGTCGGACTTCTTCGGCGGCAAGGAGCTGAACAAGAGCATCAACCCCGACGAGGCTGTCGCG	540
FN395023_L. major_ hsp70_Spain	ACGCGCATCCCGAAGGTGCAGTCCCTCGTGTCGGACTTCTTCGGCGGCAAGGAGCTGAACAAGAGCATCAACCCCGACGAGGCTGTCGCG	540
FN395022_ L. major_ hsp70_Sudan	ACGCGCATCCCGAAGGTGCAGTCCCTCGTGTCGGACTTCTTCGGCGGCAAGGAGCTGAACAAGAGCATCAACCCCGACGAGGCTGTCGCG	540

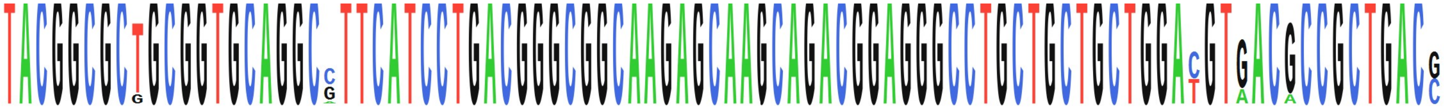

Consensus	TACGGCGCTGCGGTGCAGGCCTTCATCCTGACGGGCGGCAAGAGCAAGCAGACGGAGGGCCTGCTGCTGCTGGACGTGACGCCGCTGACG	
L. Panamensis_hsp70	TACGGCGCTGCGGTGCAGGCGTTCATCCTGACGGGCGGCAAGAGCAAGCAGACGGAGGGCCTGCTGCTGCTGGATGTGACGCCGCTGACC	630
EU599094_L. Pan...is_hsp70_Panama	TACGGCGCTGCGGTGCAGGCGTTCATCCTGACGGGCGGCAAGAGCAAGCAGACGGAGGGCCTGCTGCTGCTGGATGTGACGCCGCTGACC	630
FN395055_L. Pan...is_hsp70_Panama	TACGGCGCTGCGGTGCAGGCGTTCATCCTGACGGGCGGCAAGAGCAAGCAGACGGAGGGCCTGCTGCTGCTGGATGTGACGCCGCTGACC	630
KX573937_L. Pan...is_hsp70_Panama	TACGGCGCTGCGGTGCAGGCGTTCATCCTGACGGGCGGCAAGAGCAAGCAGACGGAGGGCCTGCTGCTGCTGGATGTGACGCCGCTGACC	630
KX573939_L. Pan...is_hsp70_Panama	TACGGCGCTGCGGTGCAGGCGTTCATCCTGACGGGCGGCAAGAGCAAGCAGACGGAGGGCCTGCTGCTGCTGGATGTGACGCCGCTGACC	630
KX573942_L. Pan...is_hsp70_Panama	TACGGCGCTGCGGTGCAGGCGTTCATCCTGACGGGCGGCAAGAGCAAGCAGACGGAGGGCCTGCTGCTGCTGGATGTGACGCCGCTGACC	630
KX573971_L. Pan...is_hsp70_Panama	TACGGCGCTGCGGTGCAGGCGTTCATCCTGACGGGCGGCAAGAGCAAGCAGACGGAGGGCCTGCTGCTGCTGGATGTGACGCCGCTGACC	630
KX573975_L. Pan...is_hsp70_Panama	TACGGCGCTGCGGTGCAGGCGTTCATCCTGACGGGCGGCAAGAGCAAGCAGACGGAGGGCCTGCTGCTGCTGGATGTGACGCCGCTGACC	630
OQ408896_L. Pan...is_hsp70_Panama	TACGGCGCTGCGGTGCAGGCGTTCATCCTGACGGGCGGCAAGAGCAAGCAGACGGAGGGCCTGCTGCTGCTGGATGTGACGCCGCTGACC	630
MW480568_L. Pa...s_hsp70_Norway	TACGGCGCTGCGGTGCAGGCGTTCATCCTGACGGGCGGCAAGAGCAAGCAGACGGAGGGCCTGCTGCTGCTGGATGTGACGCCGCTGACC	630
HF586406_L. guy...0_French Guiana	TACGGCGCTGCGGTGCAGGCGTTCATCCTGACGGGCGGCAAGAGCAAGCAGACGGAGGGCCTGCTGCTGCTGGATGTGACGCCGCTGACC	630
KX573934_L. guy...is_hsp70_Panama	TACGGCGCTGCGGTGCAGGCGTTCATCCTGACGGGCGGCAAGAGCAAGCAGACGGAGGGCCTGCTGCTGCTGGATGTGACGCCGCTGACC	630
KX574011_L. guy...is_hsp70_Panama	TACGGCGCTGCGGTGCAGGCGTTCATCCTGACGGGCGGCAAGAGCAAGCAGACGGAGGGCCTGCTGCTGCTGGATGTGACGCCGCTGACC	630
MW658405_ L. gu...70_isolate_PL11	TACGGCGCTGCGGTGCAGGCGTTCATCCTGACGGGCGGCAAGAGCAAGCAGACGGAGGGCCTGCTGCTGCTGGATGTGACGCCGCTGACC	630
MW658386_ L. gu...70_isolate_PL21	TACGGCGCTGCGGTGCAGGCGTTCATCCTGACGGGCGGCAAGAGCAAGCAGACGGAGGGCCTGCTGCTGCTGGATGTGACGCCGCTGACC	630
MW658403_ L. gu...p70_isolate_PL6	TACGGCGCTGCGGTGCAGGCGTTCATCCTGACGGGCGGCAAGAGCAAGCAGACGGAGGGCCTGCTGCTGCTGGATGTGACGCCGCTGACC	630
MG029123_L. ama...is_hsp70_Brazil	TACGGCGCTGCGGTGCAGGCCTTCATCCTGACGGGCGGCAAGAGCAAGCAGACGGAGGGCCTGCTGCTGCTGGACGTAACGCCGCTGACG	630
EU599090_L. amaz...sis_hsp70_Brazil	TACGGCGCTGCGGTGCAGGCCTTCATCCTGACGGGCGGCAAGAGCAAGCAGACGGAGGGCCTGCTGCTGCTGGACGTAACGCCGCTGACG	630
MF344859_L. ama...is_hsp70_Brazil	TACGGCGCTGCGGTGCAGGCCTTCATCCTGACGGGCGGCAAGAGCAAGCAGACGGAGGGCCTGCTGCTGCTGGACGTAACGCCGCTGACG	630
MF344857_L. ama...is_hsp70_Brazil	TACGGCGCTGCGGTGCAGGCCTTCATCCTGACGGGCGGCAAGAGCAAGCAGACGGAGGGCCTGCTGCTGCTGGACGTAACGCCGCTGACG	630
OP561801_L. amaz...is_hsp70_Bolivia	TACGGCGCTGCGGTGCAGGCCTTCATCCTGACGGGCGGCAAGAGCAAGCAGACGGAGGGCCTGCTGCTGCTGGACGTAACGCCGCTGACG	630
LN907831_L. ama...0_French Guiana	TACGGCGCTGCGGTGCAGGCCTTCATCCTGACGGGCGGCAAGAGCAAGCAGACGGAGGGCCTGCTKCTGCTGGACGTAACGCCGCTGACG	630
FN395039_L. braz...is_hsp70_Bolivia	TACGGCGCTGCGGTGCAGGCGTTCATCCTGACGGGCGGCAAGAGCAAGCAGACGGAGGGCCTGCTGCTGCTGGATGTGACGCCGCTGACC	630
FN395041_L. braz...is_hsp70_Bolivia	TACGGCGCTGCGGTGCAGGCGTTCATCCTGACGGGCGGCAAGAGCAAGCAGACGGAGGGCCTGCTGCTGCTGGATGTGACGCCGCTGACC	630
FR872760_L. braz...is_hsp70_Bolivia	TACGGCGCTGCGGTGCAGGCATTCATCCTGACGGGCGGCAAGAGCAAGCAGACGGAGGGCCTGCTGCTGCTGGATGTGACGCCGCTGACC	630
KX573945_L. bra...is_hsp70_Panama	TACGGCGCTGCGGTGCAGGCATTCATCCTGACGGGCGGCAAGAGCAAGCAGACGGAGGGCCTGCTGCTGCTGGATGTGACGCCGCTGACC	630
FR872763_L. bra...nsis_hsp70_Peru	TACGGCGCTGCGGTGCAGGCATTCATCCTGACGGGCGGCAAGAGCAAGCAGACGGAGGGCCTGCTGCTGCTGGATGTGACGCCGCTGACC	630
HF586401_L. mex...na_hsp70_Mexico	TACGGCGCTGCGGTGCAGGCCTTCATCCTGACGGGCGGCAAGAGCAAGCAGACGGAGGGCCTGCTGCTGCTGGACGTAACGCCGCTGACG	630
HF586413_L. mex...a_hsp70_Ecuador	TACGGCGCTGCGGTGCAGGCCTTCATCCTGACGGGCGGCAAGAGCAAGCAGACGGAGGGCCTGCTGCTGCTGGACGTAACGCCGCTGACG	630
FN395038_ L. mexicana_hsp70_Peru	TACGGCGCTGCGGTGCAGGCCTTCATCCTGACGGGCGGCAAGAGCAAGCAGACGGAGGGCCTGCTGCTGCTGGACGTAACGCCGCTGACG	630
EU599091_ L. me...a_hsp70_ Belize	TACGGCGCTGCGGTGCAGGCCTTCATCCTGACGGGCGGCAAGAGCAAGCAGACGGAGGGCCTGCTGCTGCTGGACGTAACGCCGCTGACG	630
MN728741_L. don...i_ hsp70_ India	TACGGCGCGGCGGTGCAGGCCTTCATCCTGACGGGCGGCAAGAGCAAGCAGACGGAGGGCCTGCTGCTGCTGGACGTGACGCCGCTGACG	630
MN728759_L. don...i_ hsp70_ Sudan	TACGGCGCGGCGGTGCAGGCCTTCATCCTGACGGGCGGCAAGAGCAAGCAGACGGAGGGCCTGCTGCTGCTGGACGTGACGCCGCTGACG	630
MN728753_L. inf...m_ hsp70_ Malta	TACGGCGCGGCGGTGCAGGCCTTCATCCTGACGGGCGGCAAGAGCAAGCAGACGGAGGGCCTGCTGCTGCTGGACGTGACGCCGCTGACG	630
MN728756_L. inf...m_ hsp70_ Sudan	TACGGCGCGGCGGTGCAGGCCTTCATCCTGACGGGCGGCAAGAGCAAGCAGACGGAGGGCCTGCTGCTGCTGGACGTGACGCCGCTGACG	630
FN395032_L. infa... hsp70_ Portugal	TACGGCGCGGCGGTGCAGGCCTTCATCCTGACGGGCGGCAAGAGCAAGCAGACGGAGGGCCTGCTGCTGCTGGACGTGACGCCGCTGACG	630
L. Tropica_hsp70	TACGGCGCTGCGGTGCAGGCCTTCATCCTGACGGGCGGCAAGAGCAAGCAGACGGAGGGCCTGCTGCTGCTGGACGTGACACCGCTGACG	630
LN907846_L. Tropica_hsp70_Israel	TACGGCGCTGCGGTGCAGGCCTTCATCCTGACGGGCGGCAAGAGCAAGCAGACGGAGGGCCTGCTGCTGCTGGACGTGACACCGCTGACG	630
MW658424_L. Tro...70_isolate_L192	TACGGCGCTGCGGTGCAGGCCTTCATCCTGACGGGCGGCAAGAGCAAGCAGACGGAGGGCCTGCTGCTGCTGGACGTGACACCGCTGACG	630
MW658401_L. Tro...p70_isolate_PL5	TACGGCGCTGCGGTGCAGGCCTTCATCCTGACGGGCGGCAAGAGCAAGCAGACGGAGGGCCTGCTGCTGCTGGACGTGACACCGCTGACG	630
MZ362273_L. Tro...p70_Afghanistan	TACGGCGCTGCGGTGCAGGCCTTCATCCTGACGGGCGGCAAGAGCAAGCAGACGGAGGGCCTGCTGCTGCTGGACGTGACACCGCTGACG	630
FN395023_ L. major_ hsp70_Spain	TACGGCGCTGCGGTGCAGGCCTTCATCCTGACGGGCGGCAAGAGCAAGCAGACGGAGGGCCTGCTGCTGCTGGACGTGACGCCGCTGACG	630
FN395022_ L. major_ hsp70_Sudan	TACGGCGCTGCGGTGCAGGCCTTCATCCTGACGGGCGGCAAGAGCAAGCAGACGGAGGGCCTGCTGCTGCTGGACGTGACGCCGCTGACG	630

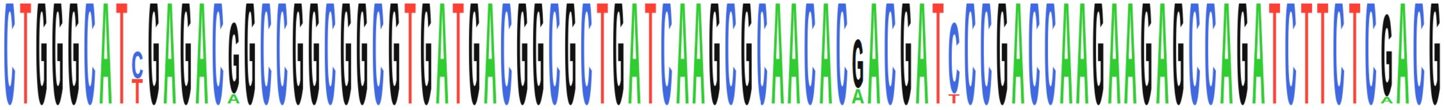

**Consensus**	CTGGGCATCGAGACGGCCGGCGGCGTGATGACGGCGCTGATCAAGCGCAACACGACGATCCCGACCAAGAAGAGCCAGATCTTCTCGACG	
L. Panamensis_hsp70	CTGGGCATTGAGACGGCCGGCGGCGTGATGACGGCGCTGATCAAGCGCAACACGACGATCCCGACCAAGAAGAGCCAGATCTTCTCGACG	720
EU599094_L. Pan...is_hsp70_Panama	CTGGGCATTGAGACGGCCGGCGGCGTGATGACGGCGCTGATCAAGCGCAACACGACGATCCCGACCAAGAAGAGCCAGATCTTCTCGACG	720
FN395055_L. Pan...is_hsp70_Panama	CTGGGCATTGAGACGGCCGGCGGCGTGATGACGGCGCTGATCAAGCGCAACACGACGATCCCGACCAAGAAGAGCCAGATCTTCTCGACG	720
KX573937_L. Pan...is_hsp70_Panama	CTGGGCATTGAGACGGCCGGCGGCGTGATGACGGCGCTGATCAAGCGCAACACGACGATCCCGACCAAGAAGAGCCAGATCTTCTCGACG	720
KX573939_L. Pan...is_hsp70_Panama	CTGGGCATTGAGACGGCCGGCGGCGTGATGACGGCGCTGATCAAGCGCAACACGACGATCCCGACCAAGAAGAGCCAGATCTTCTCGACG	720
KX573942_L. Pan...is_hsp70_Panama	CTGGGCATTGAGACGGCCGGCGGCGTGATGACGGCGCTGATCAAGCGCAACACGACGATCCCGACCAAGAAGAGCCAGATCTTCTCGACG	720
KX573971_L. Pan...is_hsp70_Panama	CTGGGCATTGAGACGGCCGGCGGCGTGATGACGGCGCTGATCAAGCGCAACACGACGATCCCGACCAAGAAGAGCCAGATCTTCTCGACG	720
KX573975_L. Pan...is_hsp70_Panama	CTGGGCATTGAGACGGCCGGCGGCGTGATGACGGCGCTGATCAAGCGCAACACGACGATCCCGACCAAGAAGAGCCAGATCTTCTCGACG	720
OQ408896_L. Pan...is_hsp70_Panama	CTGGGCATTGAGACGGCCGGCGGCGTGATGACGGCGCTGATCAAGCGCAACACGACGATCCCGACCAAGAAGAGCCAGATCTTCTCGACG	720
MW480568_L. Pa...s_hsp70_Norway	CTGGGCATTGAGACGGCCGGCGGCGTGATGACGGCGCTGATCAAGCGCAACACGACGATCCCGACCAAGAAGAGCCAGATCTTCTCGACG	720
HF586406_L. guy...0_French Guiana	CTGGGCATTGAGACGGCCGGCGGCGTGATGACGGCGCTGATCAAGCGCAACACGACGATCCCGACCAAGAAGAGCCAGATCTTCTCGACG	720
KX573934_L. guy...is_hsp70_Panama	CTGGGCATTGAGACGGCCGGCGGCGTGATGACGGCGCTGATCAAGCGCAACACGACGATCCCGACCAAGAAGAGCCAGATCTTCTCGACG	720
KX574011_L. guy...is_hsp70_Panama	CTGGGCATTGAGACGGCCGGCGGCGTGATGACGGCGCTGATCAAGCGCAACACGACGATCCCGACCAAGAAGAGCCAGATCTTCTCGACG	720
MW658405_ L. gu...70_isolate_PL11	CTGGGCATTGAGACGGCCGGCGGCGTGATGACGGCGCTGATCAAGCGCAACACGACGATCCCGACCAAGAAGAGCCAGATCTTCTCGACG	720
MW658386_ L. gu...70_isolate_PL21	CTGGGCATTGAGACGGCCGGCGGCGTGATGACGGCGCTGATCAAGCGCAACACGACGATCCCGACCAAGAAGAGCCAGATCTTCTCGACG	720
MW658403_ L. gu...p70_isolate_PL6	CTGGGCATTGAGACGGCCGGCGGCGTGATGACGGCGCTGATCAAGCGCAACACGACGATCCCGACCAAGAAGAGCCAGATCTTCTCGACG	720
MG029123_L. ama...is_hsp70_Brazil	CTGGGCATCGAGACGGCCGGCGGCGTGATGACGGCGCTGATCAAGCGCAACACAACGATCCCGACCAAGAAGAGCCAGATCTTCTCGACG	720
EU599090_L. amaz...sis_hsp70_Brazil	CTGGGCATCGAGACGGCCGGCGGCGTGATGACGGCGCTGATCAAGCGCAACACAACGATCCCGACCAAGAAGAGCCAGATCTTCTCGACG	720
MF344859_L. ama...is_hsp70_Brazil	CTGGGCATCGAGACGGCCGGCGGCGTGATGACGGCGCTGATCAAGCGCAACACAACGATCCCGACCAAGAAGAGCCAGATCTTCTCGACG	720
MF344857_L. ama...is_hsp70_Brazil	CTGGGCATCGAGACGGCCGGCGGCGTGATGACGGCGCTGATCAAGCGCAACACAACGATCCCGACCAAGAAGAGCCAGATCTTCTCGACG	720
OP561801_L. amaz...is_hsp70_Bolivia	CTGGGCATCGAGACGGCCGGCGGCGTGATGACGGCGCTGATCAAGCGCAACACAACGATCCCGACCAAGAAGAGCCAGATCTTCTCGACG	720
LN907831_L. ama...0_French Guiana	CTGGGCATCGAGACGGCCGGCGGCGTGATGACGGCGCTGATCAAGCGCAACACAACGATCCCGACCAAGAAGAGCCAGATCTTCTCGACG	720
FN395039_L. braz...is_hsp70_Bolivia	CTGGGCATTGAGACGGCCGGCGGCGTGATGACGGCGCTGATCAAGCGCAACACGACGATCCCGACCAAGAAGAGCCAGATCTTCTCGACG	720
FN395041_L. braz...is_hsp70_Bolivia	CTGGGCATTGAGACGGCCGGCGGCGTGATGACGGCGCTGATCAAGCGCAACACGACGATCCCGACCAAGAAGAGCCAGATCTTCTCGACG	720
FR872760_L. braz...is_hsp70_Bolivia	CTGGGCATTGAGACGGCCGGCGGCGTGATGACGGCGCTGATCAAGCGCAACACGACGATCCCGACCAAGAAGAGCCAGATCTTCTCGACG	720
KX573945_L. bra...is_hsp70_Panama	CTGGGCATTGAGACGGCCGGCGGCGTGATGACGGCGCTGATCAAGCGCAACACGACGATCCCGACCAAGAAGAGCCAGATCTTCTCGACG	720
FR872763_L. bra...nsis_hsp70_Peru	CTGGGCATTGAGACGGCCGGCGGCGTGATGACGGCGCTGATCAAGCGCAACACGACGATCCCGACCAAGAAGAGCCAGATCTTCTCGACG	720
HF586401_L. mex...na_hsp70_Mexico	CTGGGCATCGAGACGGCCGGCGGCGTGATGACGGCGCTGATCAAGCGCAACACAACGATCCCGACCAAGAAGAGCCAGATCTTCTCGACG	720
HF586413_L. mex...a_hsp70_Ecuador	CTGGGCATCGAGACGGCCGGCGGCGTGATGACGGCGCTGATCAAGCGCAACACAACGATCCCGACCAAGAAGAGCCAGATCTTCTCGACG	720
FN395038_ L. mexicana_hsp70_Peru	CTGGGCATCGAGACGGCCGGCGGCGTGATGACGGCGCTGATCAAGCGCAACACAACGATCCCGACCAAGAAGAGCCAGATCTTCTCGACG	720
EU599091_ L. me...a_hsp70_ Belize	CTGGGCATCGAGACGGCCGGCGGCGTGATGACGGCGCTGATCAAGCGCAACACAACGATCCCGACCAAGAAGAGCCAGATCTTCTCGACG	720
MN728741_L. don...i_ hsp70_ India	CTGGGCATCGAGACGGCCGGCGGCGTGATGACGGCGCTGATCAAGCGCAACACGACGATTCCGACCAAGAAGAGCCAGATCTTCTCGACG	720
MN728759_L. don...i_ hsp70_ Sudan	CTGGGCATCGAGACGGCCGGCGGCGTGATGACGGCGCTGATCAAGCGCAACACGACGATTCCGACCAAGAAGAGCCAGATCTTCTCGACG	720
MN728753_L. inf...m_ hsp70_ Malta	CTGGGCATCGAGACGGCCGGCGGCGTGATGACGGCGCTGATCAAGCGCAACACGACGATTCCGACCAAGAAGAGCCAGATCTTCTCGACG	720
MN728756_L. inf...m_ hsp70_ Sudan	CTGGGCATCGAGACGGCCGGCGGCGTGATGACGGCGCTGATCAAGCGCAACACGACGATTCCGACCAAGAAGAGCCAGATCTTCTCGACG	720
FN395032_L. infa... hsp70_ Portugal	CTGGGCATCGAGACGGCCGGCGGCGTGATGACGGCGCTGATCAAGCGCAACACGACGATTCCGACCAAGAAGAGCCAGATCTTCTCGACG	720
L. Tropica_hsp70	CTGGGCATCGAGACAGCCGGCGGCGTGATGACGGCGCTGATCAAGCGCAACACGACGATCCCGACCAAGAAGAGCCAGATCTTCTCAACG	720
LN907846_L. Tropica_hsp70_Israel	CTGGGCATCGAGACAGCCGGCGGCGTGATGACGGCGCTGATCAAGCGCAACACGACGATCCCGACCAAGAAGAGCCAGATCTTCTCAACG	720
MW658424_L. Tro...70_isolate_L192	CTGGGCATCGAGACAGCCGGCGGCGTGATGACGGCGCTGATCAAGCGCAACACGACGATCCCGACCAAGAAGAGCCAGATCTTCTCAACG	720
MW658401_L. Tro...p70_isolate_PL5	CTGGGCATCGAGACAGCCGGCGGCGTGATGACGGCGCTGATCAAGCGCAACACGACGATCCCGACCAAGAAGAGCCAGATCTTCTCRACG	720
MZ362273_L. Tro...p70_Afghanistan	CTGGGCATCGAGACAGCCGGCGGCGTGATGACGGCGCTGATCAAGCGCAACACGACGATCCCGACCAAGAAGAGCCAGATCTTCTCRACG	720
FN395023_L. major_ hsp70_Spain	CTGGGCATCGAGACGGCCGGCGGCGTGATGACGGCGCTGATCAAGCGCAACACGACGATCCCGACCAAGAAGAGCCAGATCTTCTCGACG	720
FN395022_ L. major_ hsp70_Sudan	CTGGGCATCGAGACGGCCGGCGGCGTGATGACGGCGCTGATCAAGCGCAACACGACGATCCCGACCAAGAAGAGCCAGATCTTCTCGACG	720

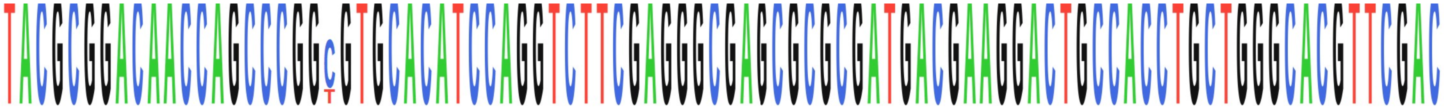

**Consensus**	TACGCGGACAACCAGCCCGGCGTGCACATCCAGGTCTTCGAGGGCGAGCGCGATGACGAAGGACTGCCACCTGCTGGGCACGTTCGAC	
L. Panamensis_hsp70	TACGCGGACAACCAGCCCGGCGTGCACATCCAGGTCTTCGAGGGCGAGCGCGATGACGAAGGACTGCCACCTGCTGGGCACGTTCGAC	810
EU599094_L. Pan…is_hsp70_Panama	TACGCGGACAACCAGCCCGGCGTGCACATCCAGGTCTTCGAGGGCGAGCGCGATGACGAAGGACTGCCACCTGCTGGGCACGTTCGAC	810
FN395055_L. Pan…is_hsp70_Panama	TACGCGGACAACCAGCCCGGCGTGCACATCCAGGTCTTCGAGGGCGAGCGCGATGACGAAGGACTGCCACCTGCTGGGCACGTTCGAC	810
KX573937_L. Pan…is_hsp70_Panama	TACGCGGACAACCAGCCCGGCGTGCACATCCAGGTCTTCGAGGGCGAGCGCGATGACGAAGGACTGCCACCTGCTGGGCACGTTCGAC	810
KX573939_L. Pan…is_hsp70_Panama	TACGCGGACAACCAGCCCGGCGTGCACATCCAGGTCTTCGAGGGCGAGCGCGATGACGAAGGACTGCCACCTGCTGGGCACGTTCGAC	810
KX573942_L. Pan…is_hsp70_Panama	TACGCGGACAACCAGCCCGGCGTGCACATCCAGGTCTTCGAGGGCGAGCGCGATGACGAAGGACTGCCACCTGCTGGGCACGTTCGAC	810
KX573971_L. Pan…is_hsp70_Panama	TACGCGGACAACCAGCCCGGCGTGCACATCCAGGTCTTCGAGGGCGAGCGCGATGACGAAGGACTGCCACCTGCTGGGCACGTTCGAC	810
KX573975_L. Pan…is_hsp70_Panama	TACGCGGACAACCAGCCCGGCGTGCACATCCAGGTCTTCGAGGGCGAGCGCGATGACGAAGGACTGCCACCTGCTGGGCACGTTCGAC	810
OQ408896_L. Pan…is_hsp70_Panama	TACGCGGACAACCAGCCCGGCGTGCACATCCAGGTCTTCGAGGGCGAGCGCGATGACGAAGGACTGCCACCTGCTGGGCACGTTCGAC	810
MW480568_L. Pa…s_hsp70_Norway	TACGCGGACAACCAGCCCGGCGTGCACATCCAGGTCTTCGAGGGCGAGCGCGATGACGAAGGACTGCCACCTGCTGGGCACGTTCGAC	810
HF586406_L. guy…0_French Guiana	TACGCGGACAACCAGCCCGGCGTGCACATCCAGGTCTTCGAGGGCGAGCGCGATGACGAAGGACTGCCACCTGCTGGGCACGTTCGAC	810
KX573934_L. guy…is_hsp70_Panama	TACGCGGACAACCAGCCCGGCGTGCACATCCAGGTCTTCGAGGGCGAGCGCGATGACGAAGGACTGCCACCTGCTGGGCACGTTCGAC	810
KX574011_L. guy…is_hsp70_Panama	TACGCGGACAACCAGCCCGGCGTGCACATCCAGGTCTTCGAGGGCGAGCGCGATGACGAAGGACTGCCACCTGCTGGGCACGTTCGAC	810
MW658405_ L. gu…70_isolate_PL11	TACGCGGACAACCAGCCCGGCGTGCACATCCAGGTCTTCGAGGGCGAGCGCGATGACGAAGGACTGCCACCTGCTGGGCACGTTCGAC	810
MW658386_ L. gu…70_isolate_PL21	TACGCGGACAACCAGCCCGGCGTGCACATCCAGGTCTTCGAGGGCGAGCGCGATGACGAAGGACTGCCACCTGCTGGGCACGTTCGAC	810
MW658403_ L. gu…p70_isolate_PL6	TACGCGGACAACCAGCCCGGCGTGCACATCCAGGTCTTCGAGGGCGAGCGCGATGACGAAGGACTGCCACCTGCTGGGCACGTTCGAC	810
MG029123_L. ama…is_hsp70_Brazil	TACGCGGACAACCAGCCCGGTGTGCACATCCAGGTCTTCGAGGGCGAGCGCGATGACGAAGGACTGCCACCTGCTGGGCACGTTCGAC	810
EU599090_L. amaz…sis_hsp70_Brazil	TACGCGGACAACCAGCCCGGTGTGCACATCCAGGTCTTCGAGGGCGAGCGCGATGACGAAGGACTGCCACCTGCTGGGCACGTTCGAC	810
MF344859_L. ama…is_hsp70_Brazil	TACGCGGACAACCAGCCCGGTGTGCACATCCAGGTCTTCGAGGGCGAGCGCGATGACGAAGGACTGCCACCTGCTGGGCACGTTCGAC	810
MF344857_L. ama…is_hsp70_Brazil	TACGCGGACAACCAGCCCGGTGTGCACATCCAGGTCTTCGAGGGCGAGCGCGATGACGAAGGACTGCCACCTGCTGGGCACGTTCGAC	810
OP561801_L. amaz…is_hsp70_Bolivia	TACGCGGACAACCAGCCCGGTGTGCACATCCAGGTCTTCGAGGGCGAGCGCGATGACGAAGGACTGCCACCTGCTGGGCACGTTCGAC	810
LN907831_L. ama…0_French Guiana	TACGCGGACAACCAGCCCGGTGTGCACATCCAGGTCTTCGAGGGCGAGCGCGATGACGAAGGACTGCCACCTGCTGGGCACGTTCGAC	810
FN395039_L. braz…is_hsp70_Bolivia	TACGCGGACAACCAGCCCGGCGTGCACATCCAGGTCTTCGAGGGCGAGCGCGATGACGAAGGACTGCCACCTGCTGGGCACGTTCGAC	810
FN395041_L. braz…is_hsp70_Bolivia	TACGCGGACAACCAGCCCGGCGTGCACATCCAGGTCTTCGAGGGCGAGCGCGATGACGAAGGACTGCCACCTGCTGGGCACGTTCGAC	810
FR872760_L. braz…is_hsp70_Bolivia	TACGCGGACAACCAGCCCGGCGTGCACATCCAGGTCTTCGAGGGCGAGCGCGATGACGAAGGACTGCCACCTGCTGGGCACGTTCGAC	810
KX573945_L. bra…is_hsp70_Panama	TACGCGGACAACCAGCCCGGCGTGCACATCCAGGTCTTCGAGGGCGAGCGCGATGACGAAGGACTGCCACCTGCTGGGCACGTTCGAC	810
FR872763_L. bra…nsis_hsp70_Peru	TACGCGGACAACCAGCCCGGCGTGCACATCCAGGTCTTCGAGGGCGAGCGCGATGACGAAGGACTGCCACCTGCTGGGCACGTTCGAC	810
HF586401_L. mex…na_hsp70_Mexico	TACGCGGACAACCAGCCCGGTGTGCACATCCAGGTCTTCGAGGGCGAGCGCGATGACGAAGGACTGCCACCTGCTGGGCACGTTCGAC	810
HF586413_L. mex…a_hsp70_Ecuador	TACGCGGACAACCAGCCCGGTGTGCACATCCAGGTCTTCGAGGGCGAGCGCGATGACGAAGGACTGCCACCTGCTGGGCACGTTCGAC	810
FN395038_ L. mexicana_hsp70_Peru	TACGCGGACAACCAGCCCGGTGTGCACATCCAGGTCTTCGAGGGCGAGCGCGATGACGAAGGACTGCCACCTGCTGGGCACGTTCGAC	810
EU599091_ L. me…a_hsp70_ Belize	TACGCGGACAACCAGCCCGGTGTGCACATCCAGGTCTTCGAGGGCGAGCGCGATGACGAAGGACTGCCACCTGCTGGGCACGTTCGAC	810
MN728741_L. don…i_ hsp70_ India	TACGCGGACAACCAGCCCGGCGTGCACATCCAGGTCTTCGAGGGCGAGCGCGATGACGAAGGACTGCCACCTGCTGGGCACGTTCGAC	810
MN728759_L. don…i_ hsp70_ Sudan	TACGCGGACAACCAGCCCGGCGTGCACATCCAGGTCTTCGAGGGCGAGCGCGATGACGAAGGACTGCCACCTGCTGGGCACGTTCGAC	810
MN728753_L. inf…m_ hsp70_ Malta	TACGCGGACAACCAGCCCGGCGTGCACATCCAGGTCTTCGAGGGCGAGCGCGATGACGAAGGACTGCCACCTGCTGGGCACGTTCGAC	810
MN728756_L. inf…m_ hsp70_ Sudan	TACGCGGACAACCAGCCCGGCGTGCACATCCAGGTCTTCGAGGGCGAGCGCGATGACGAAGGACTGCCACCTGCTGGGCACGTTCGAC	810
FN395032_L. infa… hsp70_ Portugal	TACGCGGACAACCAGCCCGGCGTGCACATCCAGGTCTTCGAGGGCGAGCGCGATGACGAAGGACTGCCACCTGCTGGGCACGTTCGAC	810
L. Tropica_hsp70	TACGCGGACAACCAGCCCGGCGTGCACATCCAGGTCTTCGAGGGCGAGCGCGATGACGAAGGACTGCCACCTGCTGGGCACGTTCGAC	810
LN907846_L. Tropica_hsp70_Israel	TACGCGGACAACCAGCCCGGCGTGCACATCCAGGTCTTCGAGGGCGAGCGCGATGACGAAGGACTGCCACCTGCTGGGCACGTTCGAC	810
MW658424_L. Tro…70_isolate_L192	TACGCGGACAACCAGCCCGGCGTGCACATCCAGGTCTTCGAGGGCGAGCGCGATGACGAAGGACTGCCACCTGCTGGGCACGTTCGAC	810
MW658401_L. Tro…p70_isolate_PL5	TACGCGGACAACCAGCCCGGCGTGCACATCCAGGTCTTCGAGGGCGAGCGCGATGACGAAGGACTGCCACCTGCTGGGCACGTTCGAC	810
MZ362273_L. Tro…p70_Afghanistan	TACGCGGACAACCAGCCCGGCGTGCACATCCAGGTCTTCGAGGGCGAGCGCGATGACGAAGGACTGCCACCTGCTGGGCACGTTCGAC	810
FN395023_L. major_ hsp70_Spain	TACGCGGACAACCAGCCCGGCGTGCACATCCAGGTCTTCGAGGGCGAGCGCGATGACGAAGGACTGCCACCTGCTGGGCACGTTCGAC	810
FN395022_ L. major_ hsp70_Sudan	TACGCGGACAACCAGCCCGGCGTGCACATCCAGGTCTTCGAGGGCGAGCGCGATGACGAAGGACTGCCACCTGCTGGGCACGTTCGAC	810

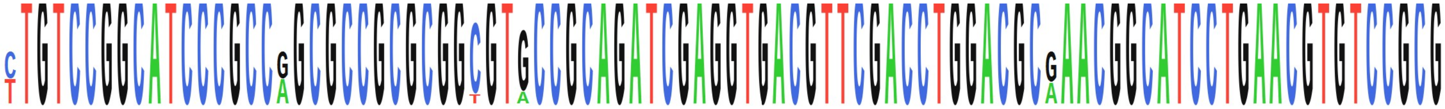

Sequence	Alignment	Position
**Consensus**	CTGTCCGGCATCCCGCCGGCGCCGCGCGGCGTGCCGCAGATCGAGGTGACGTTCGACCTGGACGCGAACGGCATCCTGAACGTGTCCGCG	
L. Panamensis_hsp70	TTGTCCGGCATCCCGCCAGCGCCGCGCGGCGTGCCGCAGATCGAGGTGACGTTCGACCTGGACGCGAACGGCATCCTGAACGTGTCCGCG	900
EU599094_L. Pan...is_hsp70_Panama	TTGTCCGGCATCCCGCCAGCGCCGCGCGGCGTGCCGCAGATCGAGGTGACGTTCGACCTGGACGCGAACGGCATCCTGAACGTGTCCGCG	900
FN395055_L. Pan...is_hsp70_Panama	TTGTCCGGCATCCCGCCAGCGCCGCGCGGCGTGCCGCAGATCGAGGTGACGTTCGACCTGGACGCGAACGGCATCCTGAACGTGTCCGCG	900
KX573937_L. Pan...is_hsp70_Panama	TTGTCCGGCATCCCGCCAGCGCCGCGCGGCGTGCCGCAGATCGAGGTGACGTTCGACCTGGACGCGAACGGCATCCTGAACGTGTCCGCG	900
KX573939_L. Pan...is_hsp70_Panama	TTGTCCGGCATCCCGCCAGCGCCGCGCGGCGTGCCGCAGATCGAGGTGACGTTCGACCTGGACGCGAACGGCATCCTGAACGTGTCCGCG	900
KX573942_L. Pan...is_hsp70_Panama	TTGTCCGGCATCCCGCCAGCGCCGCGCGGCGTGCCGCAGATCGAGGTGACGTTCGACCTGGACGCGAACGGCATCCTGAACGTGTCCGCG	900
KX573971_L. Pan...is_hsp70_Panama	TTGTCCGGCATCCCGCCAGCGCCGCGCGGCGTGCCGCAGATCGAGGTGACGTTCGACCTGGACGCGAACGGCATCCTGAACGTGTCCGCG	900
KX573975_L. Pan...is_hsp70_Panama	TTGTCCGGCATCCCGCCAGCGCCGCGCGGCGTGCCGCAGATCGAGGTGACGTTCGACCTGGACGCGAACGGCATCCTGAACGTGTCCGCG	900
OQ408896_L. Pan...is_hsp70_Panama	TTGTCCGGCATCCCGCCAGCGCCGCGCGGCGTGCCGCAGATCGAGGTGACGTTCGACCTGGACGCGAACGGCATCCTGAACGTGTCCGCG	900
MW480568_L. Pa...s_hsp70_Norway	TTGTCCGGCATCCCGCCAGCGCCGCGCGGCGTGCCGCAGATCGAGGTGACGTTCGACCTGGACGCGAACGGCATCCTGAACGTGTCCGCG	900
HF586406_L. guy...0_French Guiana	TTGTCCGGCATCCCGCCAGCGCCGCGCGGCGTGCCGCAGATCGAGGTGACGTTCGACCTGGACGCGAACGGCATCCTGAACGTGTCCGCG	900
KX573934_L. guy...is_hsp70_Panama	TTGTCCGGCATCCCGCCAGCGCCGCGCGGCGTGCCGCAGATCGAGGTGACGTTCGACCTGGACGCGAACGGCATCCTGAACGTGTCCGCG	900
KX574011_L. guy...is_hsp70_Panama	TTGTCCGGCATCCCGCCAGCGCCGCGCGGCGTGCCGCAGATCGAGGTGACGTTCGACCTGGACGCGAACGGCATCCTGAACGTGTCCGCG	900
MW658405_ L. gu...70_isolate_PL11	TTGTCCGGCATCCCGCCAGCGCCGCGCGGCGTGCCGCAGATCGAGGTGACGTTCGACCTGGACGCGAACGGCATCCTGAACGTGTCCGCG	900
MW658386_ L. gu...70_isolate_PL21	TTGTCCGGCATCCCGCCAGCGCCGCGCGGCGTGCCGCAGATCGAGGTGACGTTCGACCTGGACGCGAACGGCATCCTGAACGTGTCCGCG	900
MW658403_ L. gu...p70_isolate_PL6	TTGTCCGGCATCCCGCCAGCGCCGCGCGGCGTGCCGCAGATCGAGGTGACGTTCGACCTGGACGCGAACGGCATCCTGAACGTGTCCGCG	900
MG029123_L. ama...is_hsp70_Brazil	CTGTCCGGCATCCCGCCGGCGCCGCGCGGCGTGCCGCAGATCGAGGTGACGTTCGACCTGGACGCAAACGGCATCCTGAACGTGTCCGCG	900
EU599090_L. amaz...sis_hsp70_Brazil	CTGTCCGGCATCCCGCCGGCGCCGCGCGGCGTGCCGCAGATCGAGGTGACGTTCGACCTGGACGCAAACGGCATCCTGAACGTGTCCGCG	900
MF344859_L. ama...is_hsp70_Brazil	CTGTCCGGCATCCCGCCGGCGCCGCGCGGCGTGCCGCAGATCGAGGTGACGTTCGACCTGGACGCAAACGGCATCCTGAACGTGTCCGCG	900
MF344857_L. ama...is_hsp70_Brazil	CTGTCCGGCATCCCGCCGGCGCCGCGCGGCGTGCCGCAGATCGAGGTGACGTTCGACCTGGACGCAAACGGCATCCTGAACGTGTCCGCG	900
OP561801_L. amaz...is_hsp70_Bolivia	CTGTCCGGCATCCCGCCGGCGCCGCGCGGCGTGCCGCAGATCGAGGTGACGTTCGACCTGGACGCAAACGGCATCCTGAACGTGTCCGCG	900
LN907831_L. ama...0_French Guiana	CTGTCCGGCATCCCGCCGGCGCCGCGCGGCGTGCCGCAGATCGAGGTGACGTTCGACCTGGACGCAAACGGCATCCTGAACGTGTCCGCG	900
FN395039_L. braz...is_hsp70_Bolivia	TTGTCCGGCATCCCGCCAGCGCCGCGCGGCGTGCCGCAGATCGAGGTGACGTTCGACCTGGACGCGAACGGCATCCTGAACGTGTCCGCG	900
FN395041_L. braz...is_hsp70_Bolivia	TTGTCCGGCATCCCGCCAGCGCCGCGCGGCGTGCCGCAGATCGAGGTGACGTTCGACCTGGACGCGAACGGCATCCTGAACGTGTCCGCG	900
FR872760_L. braz...is_hsp70_Bolivia	TTGTCCGGCATCCCGCCAGCGCCGCGCGGCGTGCCGCAGATCGAGGTGACGTTCGACCTGGACGCGAACGGCATCCTGAACGTGTCCGCG	900
KX573945_L. bra...is_hsp70_Panama	TTGTCCGGCATCCCGCCAGCGCCGCGCGGCGTGCCGCAGATCGAGGTGACGTTCGACCTGGACGCGAACGGCATCCTGAACGTGTCCGCG	900
FR872763_L. bra...nsis_hsp70_Peru	TTGTCCGGCATCCCGCCAGCGCCGCGCGGCGTGCCGCAGATCGAGGTGACGTTCGACCTGGACGCGAACGGCATCCTGAACGTGTCCGCG	900
HF586401_L. mex...na_hsp70_Mexico	CTGTCCGGCATCCCGCCGGCGCCGCGCGGCGTGCCGCAGATCGAGGTGACGTTCGACCTGGACGCAAACGGCATCCTGAACGTGTCCGCG	900
HF586413_L. mex...a_hsp70_Ecuador	CTGTCCGGCATCCCGCCGGCGCCGCGCGGCGTGCCGCAGATCGAGGTGACGTTCGACCTGGACGCAAACGGCATCCTGAACGTGTCCGCG	900
FN395038_ L. mexicana_hsp70_Peru	CTGTCCGGCATCCCGCCGGCGCCGCGCGGCGTGCCGCAGATCGAGGTGACGTTCGACCTGGACGCAAACGGCATCCTGAACGTGTCCGCG	900
EU599091_ L. me...a_hsp70_ Belize	CTGTCCGGCATCCCGCCGGCGCCGCGCGGCGTGCCGCAGATCGAGGTGACGTTCGACCTGGACGCAAACGGCATCCTGAACGTGTCCGCG	900
MN728741_L. don...i_ hsp70_ India	CTGTCCGGCATCCCGCCGGCGCCGCGCGGTGTGCCGCAGATCGAGGTGACGTTCGACCTGGACGCAAACGGCATCCTGAACGTGTCCGCG	900
MN728759_L. don...i_ hsp70_ Sudan	CTGTCCGGCATCCCGCCGGCGCCGCGCGGTGTGCCGCAGATCGAGGTGACGTTCGACCTGGACGCAAACGGCATCCTGAACGTGTCCGCG	900
MN728753_L. inf...m_ hsp70_ Malta	CTGTCCGGCATCCCGCCGGCGCCGCGCGGTGTGCCGCAGATCGAGGTGACGTTCGACCTGGACGCAAACGGCATCCTGAACGTGTCCGCG	900
MN728756_L. inf...m_ hsp70_ Sudan	CTGTCCGGCATCCCGCCGGCGCCGCGCGGTGTGCCGCAGATCGAGGTGACGTTCGACCTGGACGCAAACGGCATCCTGAACGTGTCCGCG	900
FN395032_L. infa... hsp70_ Portugal	CTGTCCGGCATCCCGCCGGCGCCGCGCGGTGTGCCGCAGATCGAGGTGACGTTCGACCTGGACGCAAACGGCATCCTGAACGTGTCCGCG	900
L. Tropica_hsp70	CTGTCCGGCATCCCGCCGGCGCCGCGCGGCGTACCGCAGATCGAGGTGACGTTCGACCTGGACGCGAACGGCATCCTGAACGTGTCCGCG	900
LN907846_L. Tropica_hsp70_Israel	CTGTCCGGCATCCCGCCGGCGCCGCGCGGCGTACCGCAGATCGAGGTGACGTTCGACCTGGACGCGAACGGCATCCTGAACGTGTCCGCG	900
MW658424_L. Tro...70_isolate_L192	CTGTCCGGCATCCCGCCGGCGCCGCGCGGCGTACCGCAGATCGAGGTGACGTTCGACCTGGACGCGAACGGCATCCTGAACGTGTCCGCG	900
MW658401_L. Tro...p70_isolate_PL5	CTGTCCGGCATCCCGCCGGCGCCGCGCGGCGTACCGCAGATCGAGGTGACGTTCGACCTGGACGCRAACGGCATCCTGAACGTGTCCGCG	900
MZ362273_L. Tro...p70_Afghanistan	CTGTCCGGCATCCCGCCGGCGCCGCGCGGCGTACCGCAGATCGAGGTGACGTTCGACCTGGACGCRAACGGCATCCTGAACGTGTCCGCG	900
FN395023_L. major_ hsp70_Spain	CTGTCCGGCATCCCGCCGGCGCCGCGCGGCGTACCGCAGATCGAGGTGACGTTCGACCTGGACGCAAACGGCATCCTGAACGTGTCCGCG	900
FN395022_ L. major_ hsp70_Sudan	CTGTCCGGCATCCCGCCGGCGCCGCGCGGCGTACCGCAGATCGAGGTGACGTTCGACCTGGACGCAAACGGCATCCTGAACGTGTCCGCG	900

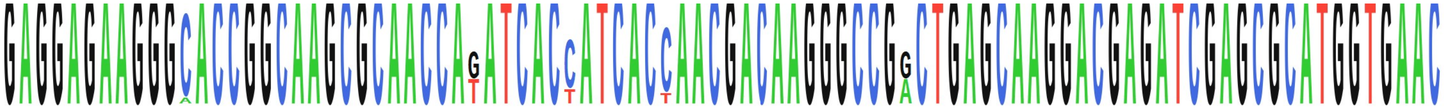

Consensus	GAGGAGAAGGGCACCGGCAAGCGCAACCAGATCACCATCACCAACGACAAGGGCCGGCTGAGCAAGGACGAGATCGAGCGCATGGTGAAC	
L. Panamensis_hsp70	GAGGAGAAGGGCACCGGCAAGCGCAACCATATCACTATCACCAACGACAAGGGCCGACTGAGCAAGGACGAGATCGAGCGCATGGTGAAC	990
EU599094_L. Pan...is_hsp70_Panama	GAGGAGAAGGGCACCGGCAAGCGCAACCATATCACTATCACCAACGACAAGGGCCGACTGAGCAAGGACGAGATCGAGCGCATGGTGAAC	990
FN395055_L. Pan...is_hsp70_Panama	GAGGAGAAGGGCACCGGCAAGCGCAACCATATCACTATCACCAACGACAAGGGCCGACTGAGCAAGGACGAGATCGAGCGCATGGTGAAC	990
KX573937_L. Pan...is_hsp70_Panama	GAGGAGAAGGGCACCGGCAAGCGCAACCATATCACTATCACCAACGACAAGGGCCGACTGAGCAAGGACGAGATCGAGCGCATGGTGAAC	990
KX573939_L. Pan...is_hsp70_Panama	GAGGAGAAGGGCACCGGCAAGCGCAACCATATCACTATCACCAACGACAAGGGCCGACTGAGCAAGGACGAGATCGAGCGCATGGTGAAC	990
KX573942_L. Pan...is_hsp70_Panama	GAGGAGAAGGGCACCGGCAAGCGCAACCATATCACTATCACCAACGACAAGGGCCGACTGAGCAAGGACGAGATCGAGCGCATGGTGAAC	990
KX573971_L. Pan...is_hsp70_Panama	GAGGAGAAGGGCACCGGCAAGCGCAACCATATCACTATCACCAACGACAAGGGCCGACTGAGCAAGGACGAGATCGAGCGCATGGTGAAC	990
KX573975_L. Pan...is_hsp70_Panama	GAGGAGAAGGGCACCGGCAAGCGCAACCATATCACTATCACCAACGACAAGGGCCGACTGAGCAAGGACGAGATCGAGCGCATGGTGAAC	990
OQ408896_L. Pan...is_hsp70_Panama	GAGGAGAAGGGCACCGGCAAGCGCAACCATATCACTATCACCAACGACAAGGGCCGACTGAGCAAGGACGAGATCGAGCGCATGGTGAAC	990
MW480568_L. Pa...s_hsp70_Norway	GAGGAGAAGGGCACCGGCAAGCGCAACCATATCACTATCACCAACGACAAGGGCCGACTGAGCAAGGACGAGATCGAGCGCATGGTGAAC	990
HF586406_L. guy...0_French Guiana	GAGGAGAAGGGCACCGGCAAGCGCAACCATATCACCATCACCAACGACAAGGGCCGACTGAGCAAGGACGAGATCGAGCGCATGGTGAAC	990
KX573934_L. guy...is_hsp70_Panama	GAGGAGAAGGGCACCGGCAAGCGCAACCATATCACCATCACCAACGACAAGGGCCGACTGAGCAAGGACGAGATCGAGCGCATGGTGAAC	990
KX574011_L. guy...is_hsp70_Panama	GAGGAGAAGGGCACCGGCAAGCGCAACCATATCACCATCACCAACGACAAGGGCCGACTGAGCAAGGACGAGATCGAGCGCATGGTGAAC	990
MW658405_ L. gu...70_isolate_PL11	GAGGAGAAGGGCACCGGCAAGCGCAACCATATCACCATCACCAACGACAAGGGCCGACTGAGCAAGGACGAGATCGAGCGCATGGTGAAC	990
MW658386_ L. gu...70_isolate_PL21	GAGGAGAAGGGCACCGGCAAGCGCAACCATATCACCATCACCAACGACAAGGGCCGACTGAGCAAGGACGAGATCGAGCGCATGGTGAAC	990
MW658403_ L. gu...p70_isolate_PL6	GAGGAGAAGGGCACCGGCAAGCGCAACCATATCACCATCACCAACGACAAGGGCCGACTGAGCAAGGACGAGATCGAGCGCATGGTGAAC	990
MG029123_L. ama...is_hsp70_Brazil	GAGGAGAAGGGCACCGGCAAGCGCAACCAGATCACCATCACTAACGACAAGGGCCGGCTGAGCAAGGACGAGATCGAGCGCATGGTGAAC	990
EU599090_L. amaz...sis_hsp70_Brazil	GAGGAGAAGGGCACCGGCAAGCGCAACCAGATCACCATCACTAACGACAAGGGCCGGCTGAGCAAGGACGAGATCGAGCGCATGGTGAAC	990
MF344859_L. ama...is_hsp70_Brazil	GAGGAGAAGGGCACCGGCAAGCGCAACCAGATCACCATCACTAACGACAAGGGCCGGCTGAGCAAGGACGAGATCGAGCGCATGGTGAAC	990
MF344857_L. ama...is_hsp70_Brazil	GAGGAGAAGGGCACCGGCAAGCGCAACCAGATCACCATCACTAACGACAAGGGCCGGCTGAGCAAGGACGAGATCGAGCGCATGGTGAAC	990
OP561801_L. amaz...is_hsp70_Bolivia	GAGGAGAAGGGCACCGGCAAGCGCAACCAGATCACCATCACTAACGACAAGGGCCGGCTGAGCAAGGACGAGATCGAGCGCATGGTGAAC	990
LN907831_L. ama...0_French Guiana	GAGGAGAAGGGCACCGGCAAGCGCAACCAGATCACCATCACYAACGACAAGGGCCGGCTGAGCAAGGACGAGATCGAGCGCATGGTGAAC	990
FN395039_L. braz...is_hsp70_Bolivia	GAGGAGAAGGGCACCGGCAAGCGCAACCATATCACCATCACCAACGACAAGGGCCGACTGAGCAAGGACGAGATCGAGCGCATGGTGAAC	990
FN395041_L. braz...is_hsp70_Bolivia	GAGGAGAAGGGCACCGGCAAGCGCAACCATATCACCATCACCAACGACAAGGGCCGACTGAGCAAGGACGAGATCGAGCGCATGGTGAAC	990
FR872760_L. braz...is_hsp70_Bolivia	GAGGAGAAGGGAACCGGCAAGCGCAACCATATCACCATCACCAACGACAAGGGCCGACTGAGCAAGGACGAGATCGAGCGCATGGTGAAC	990
KX573945_L. bra...is_hsp70_Panama	GAGGAGAAGGGAACCGGCAAGCGCAACCATATCACCATCACCAACGACAAGGGCCGACTGAGCAAGGACGAGATCGAGCGCATGGTGAAC	990
FR872763_L. bra...nsis_hsp70_Peru	GAGGAGAAGGGAACCGGCAAGCGCAACCATATCACCATCACCAACGACAAGGGCCGACTGAGCAAGGACGAGATCGAGCGCATGGTGAAC	990
HF586401_L. mex...na_hsp70_Mexico	GAGGAGAAGGGCACCGGCAAGCGCAACCAGATCACCATCACCAACGACAAGGGCCGGCTGAGCAAGGACGAGATCGAGCGCATGGTGAAC	990
HF586413_L. mex...a_hsp70_Ecuador	GAGGAGAAGGGCACCGGCAAGCGCAACCAGATCACCATCACCAACGACAAGGGCCGGCTGAGCAAGGACGAGATCGAGCGCATGGTGAAC	990
FN395038_ L. mexicana_hsp70_Peru	GAGGAGAAGGGCACCGGCAAGCGCAACCAGATCACCATCACTAACGACAAGGGCCGGCTGAGCAAGGACGAGATCGAGCGCATGGTGAAC	990
EU599091_L. me...a_hsp70_ Belize	GAGGAGAAGGGCACCGGCAAGCGCAACCAGATCACCATCACCAACGACAAGGGCCGGCTGAGCAAGGACGAGATCGAGCGCATGGTGAAC	990
MN728741_L. don...i_ hsp70_ India	GAGGAGAAGGGCACCGGCAAGCGCAACCAGATCACCATCACCAACGACAAGGGCCGGCTGAGCAAGGACGAGATCGAGCGCATGGTGAAC	990
MN728759_L. don...i_ hsp70_ Sudan	GAGGAGAAGGGCACCGGCAAGCGCAACCAGATCACCATCACCAACGACAAGGGCCGGCTGAGCAAGGACGAGATCGAGCGCATGGTGAAC	990
MN728753_L. inf...m_ hsp70_ Malta	GAGGAGAAGGGCACCGGCAAGCGCAACCAGATCACCATCACCAACGACAAGGGCCGGCTGAGCAAGGACGAGATCGAGCGCATGGTGAAC	990
MN728756_L. inf...m_ hsp70_ Sudan	GAGGAGAAGGGCACCGGCAAGCGCAACCAGATCACCATCACCAACGACAAGGGCCGGCTGAGCAAGGACGAGATCGAGCGCATGGTGAAC	990
FN395032_L. infa... hsp70_ Portugal	GAGGAGAAGGGCACCGGCAAGCGCAACCAGATCACCATCACCAACGACAAGGGCCGGCTGAGCAAGGACGAGATCGAGCGCATGGTGAAC	990
L. Tropica_hsp70	GAGGAGAAGGGCACCGGCAAGCGCAACCAGATCACCATCACCAACGACAAGGGCCGGCTGAGCAAGGACGAGATCGAGCGCATGGTGAAC	990
LN907846_L. Tropica_hsp70_Israel	GAGGAGAAGGGCACCGGCAAGCGCAACCAGATCACCATCACCAACGACAAGGGCCGGCTGAGCAAGGACGAGATCGAGCGCATGGTGAAC	990
MW658424_L. Tro...70_isolate_L192	GAGGAGAAGGGCACCGGCAAGCGCAACCAGATCACCATCACCAACGACAAGGGCCGGCTGAGCAAGGACGAGATCGAGCGCATGGTGAAC	990
MW658401_L. Tro...p70_isolate_PL5	GAGGAGAAGGGCACCGGCAAGCGCAACCAGATCACCATCACCAACGACAAGGGCCGGCTGAGCAAGGACGAGATCGAGCGCATGGTGAAC	990
MZ362273_L. Tro...p70_Afghanistan	GAGGAGAAGGGCACCGGCAAGCGCAACCAGATCACCATCACCAACGACAAGGGCCGGCTGAGCAAGGACGAGATCGAGCGCATGGTGAAC	990
FN395023_L. major_ hsp70_Spain	GAGGAGAAGGGCACCGGCAAGCGCAACCAGATCACCATCACCAACGACAAGGGCCGGCTGAGCAAGGACGAGATCGAGCGCATGGTGAAC	990
FN395022_ L. major_ hsp70_Sudan	GAGGAGAAGGGCACCGGCAAGCGCAACCAGATCACCATCACCAACGACAAGGGCCGGCTGAGCAAGGACGAGATCGAGCGCATGGTGAAC	990

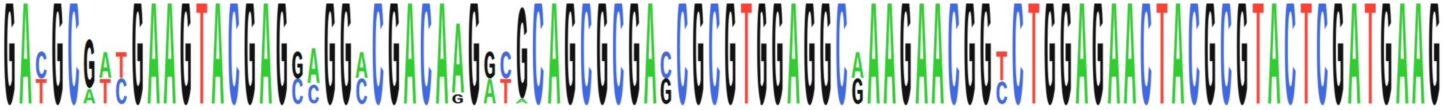

**Consensus**	GACGCGATGAAGTACGAGGAGGACGACAAGGCGCAGCGCGACCGCGTGGAGGCAAAGAACGGTCTGGAGAACTACGCGTACTCGATGAAG	
L. Panamensis_hsp70	GATGCGTCGAAGTACGAGCAGGCCGACAAGATGCAGCGCGAGCGCGTGGAGGCGAAGAACGGTCTGGAGAACTACGCGTACTCGATGAAG	1080
EU599094_L. Pan...is_hsp70_Panama	GATGCGTCGAAGTACGAGCAGGCCGACAAGATGCAGCGCGAGCGCGTGGAGGCGAAGAACGGTCTGGAGAACTACGCGTACTCGATGAAG	1080
FN395055_L. Pan...is_hsp70_Panama	GATGCGTCGAAGTACGAGCAGGCCGACAAGATGCAGCGCGAGCGCGTGGAGGCGAAGAACGGTCTGGAGAACTACGCGTACTCGATGAAG	1080
KX573937_L. Pan...is_hsp70_Panama	GATGCGTCGAAGTACGAGCAGGCCGACAAGATGCAGCGCGAGCGCGTGGAGGCGAAGAACGGTCTGGAGAACTACGCGTACTCGATGAAG	1080
KX573939_L. Pan...is_hsp70_Panama	GATGCGTCGAAGTACGAGCAGGCCGACAAGATGCAGCGCGAGCGCGTGGAGGCGAAGAACGGTCTGGAGAACTACGCGTACTCGATGAAG	1080
KX573942_L. Pan...is_hsp70_Panama	GATGCGTCGAAGTACGAGCAGGCCGACAAGATGCAGCGCGAGCGCGTGGAGGCGAAGAACGGTCTGGAGAACTACGCGTACTCGATGAAG	1080
KX573971_L. Pan...is_hsp70_Panama	GATGCGTCGAAGTACGAGCAGGCCGACAAGATGCAGCGCGAGCGCGTGGAGGCGAAGAACGGTCTGGAGAACTACGCGTACTCGATGAAG	1080
KX573975_L. Pan...is_hsp70_Panama	GATGCGTCGAAGTACGAGCAGGCCGACAAGATGCAGCGCGAGCGCGTGGAGGCGAAGAACGGTCTGGAGAACTACGCGTACTCGATGAAG	1080
OQ408896_L. Pan...is_hsp70_Panama	GATGCGTCGAAGTACGAGCAGGCCGACAAGATGCAGCGCGAGCGCGTGGAGGCGAAGAACGGTCTGGAGAACTACGCGTACTCGATGAAG	1080
MW480568_L. Pa...s_hsp70_Norway	GATGCGTCGAAGTACGAGCAGGCCGACAAGATGCAGCGCGAGCGCGTGGAGGCGAAGAACGGTCTGGAGAACTACGCGTACTCGATGAAG	1080
HF586406_L. guy...0_French Guiana	GATGCGTCGAAGTACGAGCAGGCCGACAAGATGCAGCGCGAGCGCGTGGAGGCGAAGAACGGTCTGGAGAACTACGCGTACTCGATGAAG	1080
KX573934_L. guy...is_hsp70_Panama	GATGCGTCGAAGTACGAGCAGGCCGACAAGATGCAGCGCGAGCGCGTGGAGGCGAAGAACGGTCTGGAGAACTACGCGTACTCGATGAAG	1080
KX574011_L. guy...is_hsp70_Panama	GATGCGTCGAAGTACGAGCAGGCCGACAAGATGCAGCGCGAGCGCGTGGAGGCGAAGAACGGTCTGGAGAACTACGCGTACTCGATGAAG	1080
MW658405_ L. gu...70_isolate_PL11	GATGCGTCGAAGTACGAGCAGGCCGACAAGATGCAGCGCGAGCGCGTGGAGGCGAAGAACGGTCTGGAGAACTACGCGTACTCGATGAAG	1080
MW658386_ L. gu...70_isolate_PL21	GATGCGTCGAAGTACGAGCAGGCCGACAAGATGCAGCGCGAGCGCGTGGAGGCGAAGAACGGTCTGGAGAACTACGCGTACTCGATGAAG	1080
MW658403_ L. gu...p70_isolate_PL6	GATGCGTCGAAGTACGAGCAGGCCGACAAGATGCAGCGCGAGCGCGTGGAGGCGAAGAACGGTCTGGAGAACTACGCGTACTCGATGAAG	1080
MG029123_L. ama...is_hsp70_Brazil	GACGCAATGAAGTACGAGGCGGACGACAAGGCGCAGCGCGACCGCGTGGAGGCAAAGAACGGTCTGGAGAACTACGCGTACTCGATGAAG	1080
EU599090_L. amaz...sis_hsp70_Brazil	GACGCAATGAAGTACGAGGCGGACGACAAGGCGCAGCGCGACCGCGTGGAGGCAAAGAACGGTCTGGAGAACTACGCGTACTCGATGAAG	1080
MF344859_L. ama...is_hsp70_Brazil	GACGCAATGAAGTACGAGGCGGACGACAAGGCGCAGCGCGACCGCGTGGAGGCAAAGAACGGTCTGGAGAACTACGCGTACTCGATGAAG	1080
MF344857_L. ama...is_hsp70_Brazil	GACGCAATGAAGTACGAGGCGGACGACAAGGCGCAGCGCGACCGCGTGGAGGCAAAGAACGGTCTGGAGAACTACGCGTACTCGATGAAG	1080
OP561801_L. amaz...is_hsp70_Bolivia	GACGCAATGAAGTACGAGGCGGACGACAAGGCGCAGCGCGACCGCGTGGAGGCAAAGAACGGTCTGGAGAACTACGCGTACTCGATGAAG	1080
LN907831_L. ama...0_French Guiana	GACGCAATGAAGTACGAGGCGGACGACAAGGCGCAGCGCGACCGCGTGGAGGCAAAGAACGGTCTGGAGAACTACGCGTACTCGATGAAG	1080
FN395039_L. braz...is_hsp70_Bolivia	GATGCGTCGAAGTACGAGCAGGCCGACAAGATGCAGCGCGAGCGCGTGGAGGCGAAGAACGGCCTGGAGAACTACGCGTACTCGATGAAG	1080
FN395041_L. braz...is_hsp70_Bolivia	GATGCGTCGAAGTACGAGCAGGCCGACAAGATGCAGCGCGAGCGCGTGGAGGCGAAGAACGGCCTGGAGAACTACGCGTACTCGATGAAG	1080
FR872760_L. braz...is_hsp70_Bolivia	GATGCGTCGAAGTACGAGCAGGCCGACAAGATGCAGCGCGAGCGCGTKGAGGCGAAGAACGGCCTGGAGAACTACGCGTACTCGATGAAG	1080
KX573945_L. bra...is_hsp70_Panama	GATGCGTCGAAGTACGAGCAGGCCGACAAGATGCAGCGCGAGCGCGTGGAGGCGAAGAACGGCCTGGAGAACTACGCGTACTCGATGAAG	1080
FR872763_L. bra...nsis_hsp70_Peru	GATGCGTCGAAGTACGAGCAGGCCGACAAGATGCAGCGCGAGCGCGTGGAGGCGAAGAACGGCCTGGAGAACTACGCGTACTCGATGAAG	1080
HF586401_L. mex...na_hsp70_Mexico	GACGCAATGAAGTACGAGGCGGACGACAAGGCGCAGCGCGACCGCGTGGAGGCAAAGAACGGTCTGGAGAACTACGCGTACTCGATGAAG	1080
HF586413_L. mex...a_hsp70_Ecuador	GACGCAATGAAGTACGAGGCGGACGACAAGGCGCAGCGCGACCGCGTGGAGGCAAAGAACGGCCTGGAGAACTACGCGTACTCGATGAAG	1080
FN395038_ L. mexicana_hsp70_Peru	GACGCAATGAAGTACGAGGCGGACGACAAGGCGCAGCGCGACCGCGTGGAGGCAAAGAACGGTCTGGAGAACTACGCGTACTCGATGAAG	1080
EU599091_ L. me...a_hsp70_ Belize	GACGCAATGAAGTACGAGGCGGACGACAAGGCGCAGCGCGACCGCGTGGAGGCAAAGAACGGTCTGGAGAACTACGCGTACTCGATGAAG	1080
MN728741_L. don...i_ hsp70_ India	GACGCGATGAAGTACGAGGCGGACGACAGGGCGCAGCGCGACCGCGTGGAGGCAAAGAACGGCCTGGAGAACTACGCGTACTCGATGAAG	1080
MN728759_L. don...i_ hsp70_ Sudan	GACGCGATGAAGTACGAGGCGGACGACAGGGCGCAGCGCGACCGCGTGGAGGCAAAGAACGGCCTGGAGAACTACGCGTACTCGATGAAG	1080
MN728753_L. inf...m_ hsp70_ Malta	GACGCGATGAAGTACGAGGCGGACGACAGGGCGCAGCGCGACCGCGTGGAGGCAAAGAACGGCCTGGAGAACTACGCGTACTCGATGAAG	1080
MN728756_L. inf...m_ hsp70_ Sudan	GACGCGATGAAGTACGAGGCGGACGACAGGGCGCAGCGCGACCGCGTGGAGGCAAAGAACGGCCTGGAGAACTACGCGTACTCGATGAAG	1080
FN395032_L. infa... hsp70_ Portugal	GACGCGATGAAGTACGAGGCGGACGACAGGGCGCAGCGCGACCGCGTGGAGGCAAAGAACGGCCTGGAGAACTACGCGTACTCGATGAAG	1080
L. Tropica_hsp70	GACGCGATGAAGTACGAGGAGGACGACAAGGCGCAGCGCGACCGCGTGGAGGCAAAGAACGGCCTGGAGAACTACGCGTACTCGATGAAG	1080
LN907846_L. Tropica_hsp70_Israel	GACGCGATGAAGTACGAGGAGGACGACAAGGCGCAGCGCGACCGCGTGGAGGCAAAGAACGGCCTGGAGAACTACGCGTACTCGATGAAG	1080
MW658424_L. Tro...70_isolate_L192	GACGCGATGAAGTACGAGGAGGACGACAAGGCGCAGYGCGACCGCGTGGAGGCAAAGAACGGCCTGGAGAACTACGCGTACTCGATGAAG	1080
MW658401_L. Tro...p70_isolate_PL5	GACGCGATGAAGTACGAGGAGGACGACAAGGCGCAGCGCGACCGCGTGGAGGCAAAGAACGGCCTGGAGAACTACGCGTACTCGATGAAG	1080
MZ362273_L. Tro...p70_Afghanistan	GACGCGATGAAGTACGAGGAGGACGACAAGGCGCAGCGCGACCGCGTGGAGGCAAAGAACGGCCTGGAGAACTACGCGTACTCGATGAAG	1080
FN395023_L. major_ hsp70_Spain	GACGCGATGAAGTACGAGGAGGACGACAAGGCACAGCGCGACCGCGTGGAGGCAAAGAACGGCCTGGAGAACTACGCGTACTCGATGAAG	1080
FN395022_ L. major_ hsp70_Sudan	GACGCGATGAAGTACGAGGAGGACGACAAGGCACAGCGCGACCGCGTGGAGGCAAAGAACGGCCTGGAGAACTACGCGTACTCGATGAAG	1080

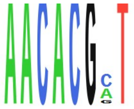

Name	Sequence	Position
**Consensus**	**AACACGCT**	
L. Panamensis_hsp70	AACACGAT	1088
EU599094_L. Pan...is_hsp70_Panama	AACACGAT	1088
FN395055_L. Pan...is_hsp70_Panama	AACACGAT	1088
KX573937_L. Pan...is_hsp70_Panama	AACACGAT	1088
KX573939_L. Pan...is_hsp70_Panama	AACACGAT	1088
KX573942_L. Pan...is_hsp70_Panama	AACACGAT	1088
KX573971_L. Pan...is_hsp70_Panama	AACACGAT	1088
KX573975_L. Pan...is_hsp70_Panama	AACACGAT	1088
OQ408896_L. Pan...is_hsp70_Panama	AACACGAT	1088
MW480568_L. Pa...s_hsp70_Norway	AACACGAT	1088
HF586406_L. guy...0_French Guiana	AACACGAT	1088
KX573934_L. guy...is_hsp70_Panama	AACACGAT	1088
KX574011_L. guy...is_hsp70_Panama	AACACGAT	1088
MW658405_ L. gu...70_isolate_PL11	AACACGAT	1088
MW658386_ L. gu...70_isolate_PL21	AACACGAT	1088
MW658403_ L. gu...p70_isolate_PL6	AACACGAT	1088
MG029123_L. ama...is_hsp70_Brazil	AACACGCT	1088
EU599090_L. amaz...sis_hsp70_Brazil	AACACGCT	1088
MF344859_L. ama...is_hsp70_Brazil	AACACGCT	1088
MF344857_L. ama...is_hsp70_Brazil	AACACGCT	1088
OP561801_L. amaz...is_hsp70_Bolivia	AACACGCT	1088
LN907831_L. ama...0_French Guiana	AACACGCT	1088
FN395039_L. braz...is_hsp70_Bolivia	AACACGGT	1088
FN395041_L. braz...is_hsp70_Bolivia	AACACGGT	1088
FR872760_L. braz...is_hsp70_Bolivia	AACACGGT	1088
KX573945_L. bra...is_hsp70_Panama	AACACGGT	1088
FR872763_L. bra...nsis_hsp70_Peru	AACACGGT	1088
HF586401_L. mex...na_hsp70_Mexico	AACACGCT	1088
HF586413_L. mex...a_hsp70_Ecuador	AACACGCT	1088
FN395038_ L. mexicana_hsp70_Peru	AACACGCT	1088
EU599091_ L. me...a_hsp70_ Belize	AACACGCT	1088
MN728741_L. don...i_ hsp70_ India	AACACGCT	1088
MN728759_L. don...i_ hsp70_ Sudan	AACACGCT	1088
MN728753_L. inf...m_ hsp70_ Malta	AACACGCT	1088
MN728756_L. inf...m_ hsp70_ Sudan	AACACGCT	1088
FN395032_L. infa... hsp70_ Portugal	AACACGCT	1088
L. Tropica_hsp70	AACACGCT	1088
LN907846_L. Tropica_hsp70_Israel	AACACGCT	1088
MW658424_L. Tro...70_isolate_L192	AACACGCT	1088
MW658401_L. Tro...p70_isolate_PL5	AACACGCT	1088
MZ362273_L. Tro...p70_Afghanistan	AACACGCT	1088
FN395023_L. major_ hsp70_Spain	AACACGCT	1088
FN395022_ L. major_ hsp70_Sudan	AACACGCT	1088

Sequence Logo: 50% GC base composition

Consensus Threshold: >50%

Compare to: the consensus

Bases that match the reference are marked with yellow highlighting.

Created: 13 Jan 2026

Last Modified: 13 Jan 2026

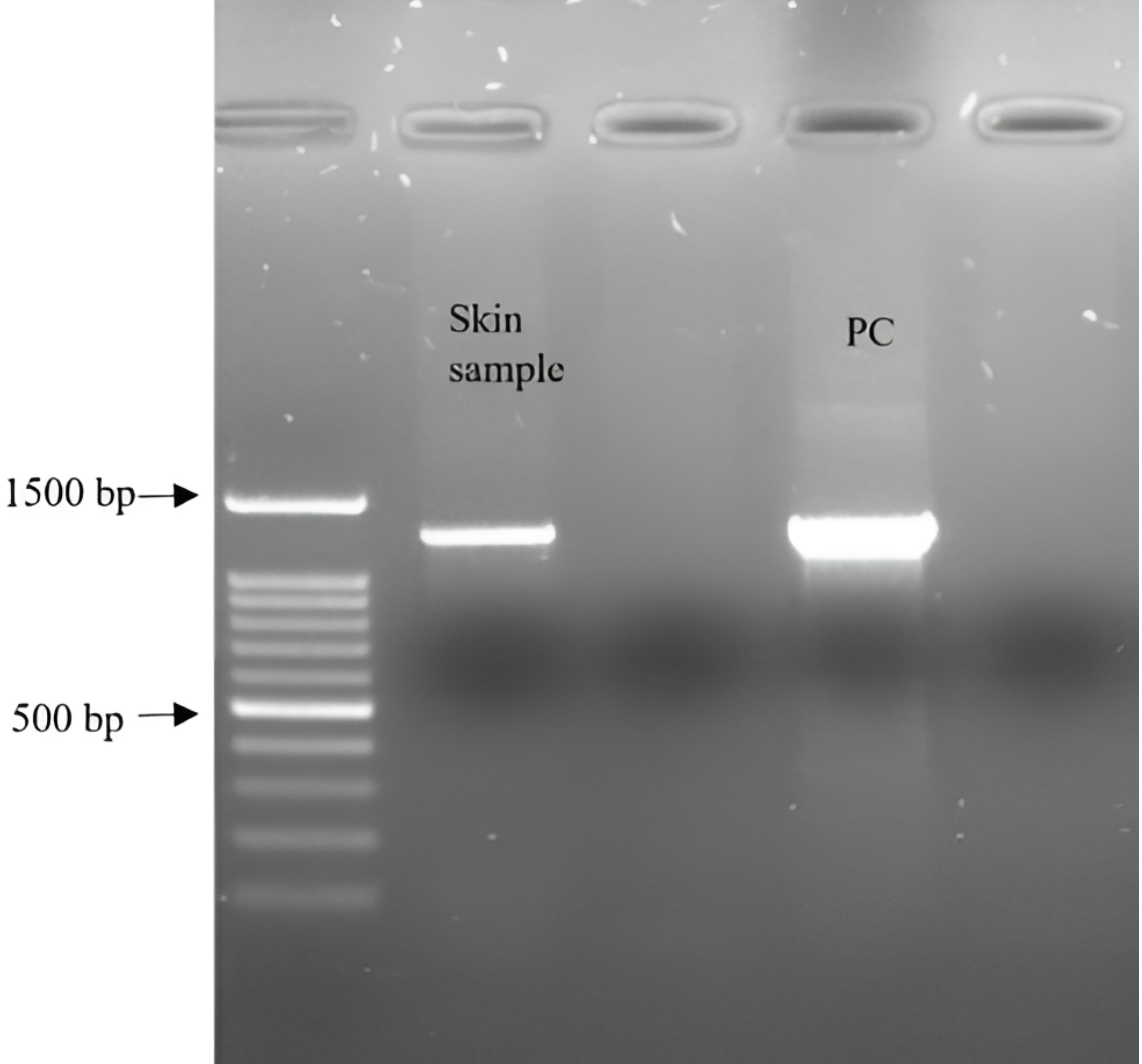

1500 bp
500 bp
Skin sample
PC

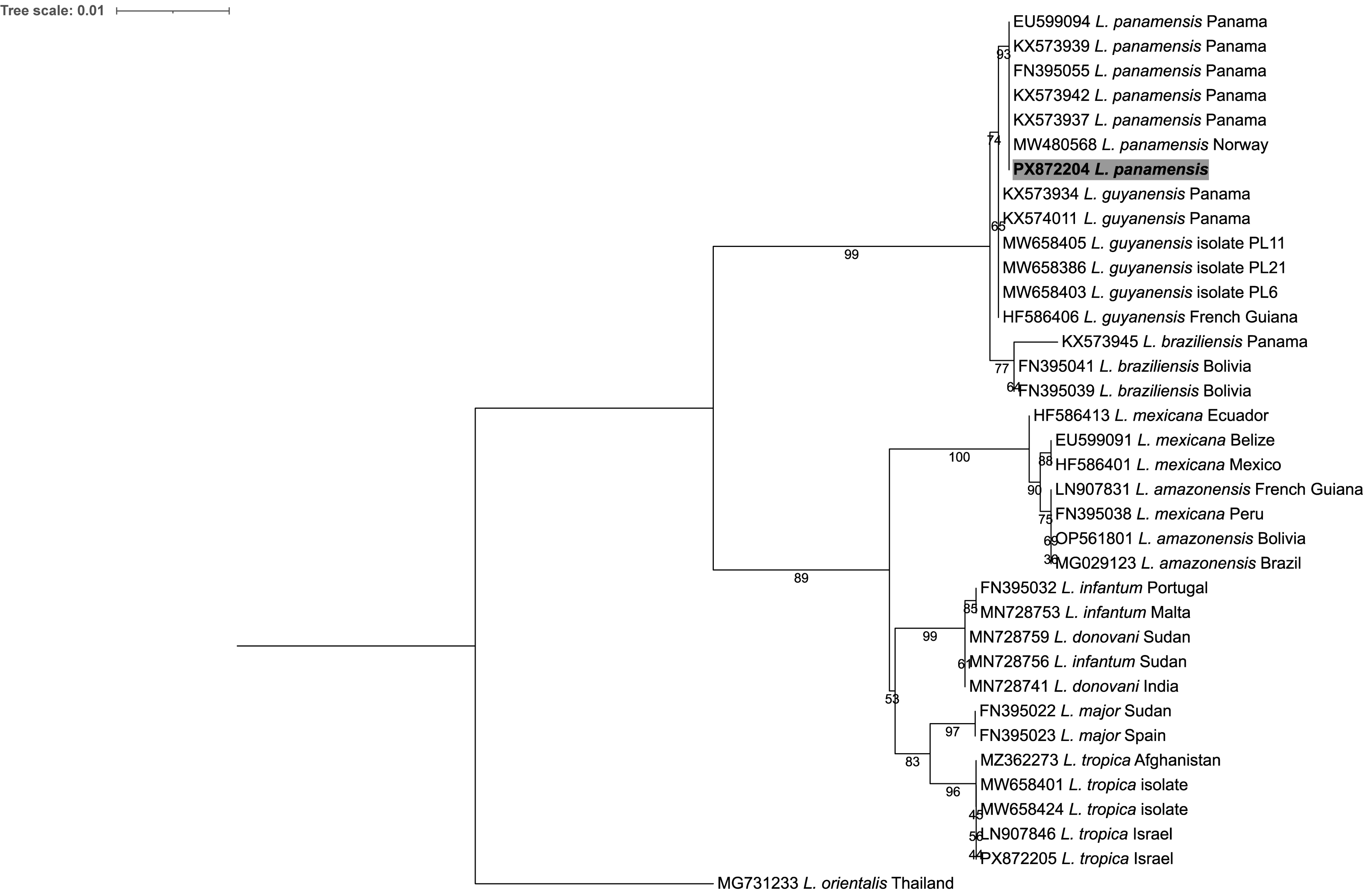

Tree scale: 0.01
EU599094 L. panamensis Panama
KX573939 L. panamensis Panama
FN395055 L. panamensis Panama
KX573942 L. panamensis Panama
KX573937 L. panamensis Panama
MW480568 L. panamensis Norway
PX872204 L. panamensis
KX573934 L. guyanensis Panama
KX574011 L. guyanensis Panama
MW658405 L. guyanensis isolate PL11
MW658386 L. guyanensis isolate PL21
MW658403 L. guyanensis isolate PL6
HF586406 L. guyanensis French Guiana
KX573945 L. braziliensis Panama
FN395041 L. braziliensis Bolivia
FN395039 L. braziliensis Bolivia
HF586413 L. mexicana Ecuador
EU599091 L. mexicana Belize
HF586401 L. mexicana Mexico
LN907831 L. amazonensis French Guiana
FN395038 L. mexicana Peru
OP561801 L. amazonensis Bolivia
MG029123 L. amazonensis Brazil
FN395032 L. infantum Portugal
MN728753 L. infantum Malta
MN728759 L. donovani Sudan
MN728756 L. infantum Sudan
MN728741 L. donovani India
FN395022 L. major Sudan
FN395023 L. major Spain
MZ362273 L. tropica Afghanistan
MW658401 L. tropica isolate
MW658424 L. tropica isolate
LN907846 L. tropica Israel
PX872205 L. tropica Israel
MG731233 L. orientalis Thailand
93
74
65
99
77
64
100
88
90
75
69
36
89
85
99
61
53
97
83
96
45
56
44
